# Supplementary material for: Carvedilol decreases hepatic vascular resistance by reducing fibrogenesis and reversing endothelial dysfunction in cirrhotic rats
Source: JHEP Rep. 2025 Nov 20;8(3):101681. doi: 10.1016/j.jhepr.2025.101681 (PMC12878606; doi:10.1016/j.jhepr.2025.101681)
Supplement: Multimedia component 1 [file mmc1.pdf]

Carvedilol decreases hepatic vascular resistance by reducing fibrogenesis and reversing endothelial dysfunction in cirrhotic rats

Yeldos Nulan, Eric Felli, Sonia-Emilia Selicean, Manuel Prampolini, Annalisa Berzigotti, Jordi Gracia-Sancho, Jaime Bosch

Table of contents

Supplementary methods………………………………………………………………....2

Fig. S1………………………………………………………………………………....6

Table S1……………………………………………………………………………... 9

Table S2……………………………………………………………………………...10

Supplementary references……………………………………………………………… 11

Supplementary methods

Animal models of cirrhosis of the liver

Female and male Sprague-Dawley rats, were acquired and maintained under standard environmental conditions at the University of Bern animal facility. The conditions included a 12-hour light/dark cycle and 10-15 air exchanges per hour. Upon arrival, the rats were acclimatized for two weeks before starting any experiments or treatments. Throughout the study, the rats had free access to chow diet and water. All experimental procedures were approved by the Bern Cantonal Ethics Committee and adhered to the Laboratory Animal Care guidelines (approval number BE382022). The model of cirrhosis and portal hypertension (PH) was based on the twice weekly for a total of 12 weeks administration of thioacetamide (TAA) (Sigma-Aldrich; Ref. No. 172502) (200 mg/kg of body weight; intraperitoneally for 12 weeks). In addition, other groups of animals received TAA for only 9 weeks, which resulted in the development of an early cirrhosis stage. Before each administration, the rats' body weights were recorded, and the TAA dosage was adjusted accordingly. Animal welfare: Animals were regularly monitored for signs of distress or discomfort, and humane endpoints were established. All invasive procedures were carried out under appropriate anesthesia, and analgesia was administered as needed. All personnel were trained in proper animal handling.

Hepatic and systemic hemodynamics

Cirrhotic rats were administered either 10 mg/kg/day carvedilol (Hexal AG, Germany), 30 mg/kg/day propranolol (Ratiopharm, Germany), or water by oral gavage for two weeks. One hour after the final dose, the rats were anesthetized with inhaled isoflurane (Girovet: 469860). Anesthesia was 5% in induction and 2% in the maintenance period. Mean arterial pressure (MAP) was measured by cannulating the femoral artery, and portal pressure (PP) was measured by cannulating the mesenteric vein, both using a heparinized P50 catheter (Portex) connected to a pressure transducer. Portal blood flow (PBF) was determined using a non-constrictive perivascular ultrasonic transit-time flow probe (Transonic Systems Inc.) placed close to the liver hilus. Pressure and flow probes were connected to a PowerLab (4SP) data recording system, and data was displayed using LabChart v5.5.6 software. Hemodynamic parameters were recorded following a 20-minute stabilization period,

collecting stable data for 2 minutes. Temperature was regulated via a heating pad (temperature maintenance of 37°C; Physitemp TCAT-2LV) with live temp control with a rectal probe. After the hemodynamic study, blood samples were collected. Finally, the animals were euthanized with a pentobarbital overdose (200 mg/kg, Nembutal), and tissue samples were harvested for molecular and histological experiments.

Isolation of liver sinusoidal endothelial cells and pharmacological treatment

LSECs were plated on a plastic plate (collagen type I pre-coated) and allowed to attach for 40 mins. After two washes with DPBS, LSECs were treated with a 10 μM propranolol, 10 μM carvedilol, or vehicle (dimethylsulfoxide (0.1%), Sigma) for another 24 h. The isolation protocol was generated by Fernández-Iglesias et.al. (1). Rats were intraperitoneally anesthetized with a combination of ketamine (100 mg/kg) and midazolam (5 mg/kg), with doses reduced by 30% for cirrhotic rats. The livers were perfused with a modified Hank's Buffer, followed by two collagenase A digestion steps: performed in situ and after liver excision. The resulting liver suspension was filtered into an ice-cold buffer through a 100 μm nylon strainer. It was then centrifuged at 60g for 5 minutes, yielding a pellet containing hepatocytes and a supernatant with non-parenchymal cells (NPCs). NPCs were further separated via a three-step OptiPrep iodixanol gradient centrifugation (0%, 13%, and 17%) into hepatic stellate cells (HSCs) and a fraction containing liver sinusoidal endothelial cells (LSECs) and Kupffer cells (KCs). LSECs were then isolated from KCs by exploiting non-specific adhesion.

Cell culture

Freshly isolated LSECs were cultured in RPMI 1640 Medium (ThermoFisher: 21875034) supplemented with 10% Fetal Bovine Serum (FBS, Gibco: A4766801), 1% antibiotic-antimycotic (Sigma: A5955), 50 μg/mL endothelial cell growth supplement (ECGS, Merck: 02-102), and 100 μg/mL heparin (Sigma: H-3393). Freshly isolated HSCs were maintained in Iscove's Modified Dulbecco's Medium (IMDM, ThermoFisher: 12440053) with 10% FBS and 1% antibiotic-antimycotic. HUVECs, purchased from Lonza (C2519A, Switzerland), were cultured in Medium 199 (Sigma-Aldrich: M4530) containing 20% FBS, 1% antibiotic-antimycotic, 50 μg/mL ECGS, and 100 μg/mL heparin. The LX-2 human hepatic stellate cell line was obtained from the Liver Vascular Biology Research Group, IDIBAPS Biomedical Research Institute and cultured in Dulbecco's Modified Eagle's Medium/Nutrient Mixture F-12 (DMEM, Gibco:

11330032) with 10% FBS and 1% antibiotic-antimycotic. All cell types were incubated at 37°C in a humid atmosphere (95%) with 5% CO_2.

Metabolic activity of Carvedilol and Propranolol in vitro

Cells in the exponential growth phase were seeded into 96-well plates with 100 µl of media per well and incubated for 24 hours. Subsequently, the cells were treated with various concentrations of carvedilol (0–100 µmol; Sigma: C3993) or propranolol (0–1000 µmol; Selleckchem: S4076) for 24 hours. After treatment, wells were gently washed twice with sterile PBS to remove residual drug and serum that could interfere with the assay. On the third day, 10 µl of MTT solution (5 mg/ml) was added to each well and incubated for 4 hours. Following incubation, the supernatant was carefully removed, and 100 µl of dimethylsulfoxide (DMSO; Sigma, Cat. No. 41647) was added to dissolve the formazan crystals. The DMSO concentration was maintained at 0.1%, and the total reagent volume was optimized for compatibility with our plate reader. After 15 minutes of incubation at room temperature in the dark, absorbance was measured at 595 nm using a BioTek Microplate Spectrophotometer (data not shown).

Cell proliferation assay

The proliferation profile of the human hepatic stellate cell line (LX2) was evaluated by the 5-bromo-2'-deoxyuridine (BrdU) incorporation assay. Briefly, cells were cultured in a 6-well plate with 1 ml media for 24 h. After that, cells were treated with or without carvedilol (10 µM) for 24 h. The culture medium was replaced with the BrdU labeling solution (Thermo: B23151) for 2 h in the incubator, followed by washing twice with PBS. Then, cells are fixed with 4% paraformaldehyde (PFA) for 15 mins at RT and washed three times with PBS. Permeabilization was achieved by adding 1ml 0.1% triton X-100 (Sigma) in RT for 20 mins. Subsequently, cells were treated with 1 ml of 1N hydrochloric acid (HCL) for 10 mins on ice, followed by 1 ml of 2N HCl for 10 mins at RT. Acid treatment was neutralized by incubating the cells with 1 ml of phosphate/citric acid buffer (pH 7.4, 182 mL of 0.2 M Na_2HPO_4 + 18 mL 0.1 M citric acid) for 10 mins at RT. The cells were washed three times with 0.1% triton X-100 (Sigma). To detect incorporated BrdU, cells were incubated with 1ml of BrdU antibody (Thermo: MA3-071, 1:100) at 4°C overnight. After washing three times with 0.1% triton X-100 (Sigma), cells were incubated with a fluorescently labeled secondary antibody for 1 hour at RT (Thermo: A21422, 2µg/ml). The cells were washed with PBS and then

for imaging in EVOS 5000 with a 10× objective in the Red Fluorescent Protein (RFP) channel. BrdU-positive cells were discriminated over DAPI channels and normalized with the Vehicle group. Quantification was made with ImageJ. Cell seeding density was 30,000/cm^2.

Cell contraction

Cell contraction assays of HSCs and LX2 cells were done in collagen lattices (collagen I rat tail; ThermoFisher: A1048301). Briefly, freshly isolated HSCs were cultured in a plastic flask for 5 days to reach a fully activated morphology. Then, 24-well culture plates were pre-incubated with 1% Bovine Serum Albumin (BSA, Sigma: A7906) in PBS, after which they were filled with a mixture of collagen I (1.3 mg/ml) and HSCs (1.5×10^5 cells/ml). After collagen gel solidification, serum-free media containing 10 µM carvedilol or propranolol was added for 24 h incubation. Cell contraction was induced by adding 10% FBS for 48 h and photographed at 1, 24, and 48 h. In another set of experiments, 500 µM of the α1-adrenergic receptor agonist methoxamine (Merck: M6524) was used to accelerate the cell contraction and the inhibitory activity of carvedilol and propranolol (both 10 µM) was analyzed in photographs at 1 h.

Superoxide detection

Superoxide (O^{2-}) levels were assessed using the oxidative fluorescent dye Dihydroethidium (DHE, 10 µM, Sigma, D7008) in liver-frozen sections, HUVECs, and LX2 cells. Cirrhotic liver tissue frozen at -80°C was sectioned into 10-micrometer slices using the Leica Cryostat CM1950 system. The tissue sections were loaded with 10 µM DHE and incubated at RT for 30 minutes. Slides were then imaged with fluorescent aqueous mounting medium (Dako; S302380-2). For the cell lines, the culture medium was removed, and the cells were rinsed twice with PBS. Subsequently, 10 µM DHE was added, and the cells were incubated for 30 minutes in an incubator. After incubation, the dye solution was replaced with PBS, and images were immediately captured. All fluorescent images were acquired using an EVOS 5000 microscope with a 10x objective. Quantitative analysis of the fluorescent signal was performed using ImageJ software version 1.53.

Immunostaining

Liver tissue for immunohistochemistry was fixed in 4% formaldehyde (Sigma), embedded in paraffin, and sectioned. Dewaxing the tissue with Xylene (2 times,

5mins) and series ethanol dilution. Heated-EDTA-Tris buffer (PH=9) was used to reveal the antigen and rinsed with distilled water two times. Unspecific binding was blocked with 3% goat serum for 1 h. Liver sections were incubated with primary antibody against alpha-smooth muscle actin (α-SMA) (Thermo; 14976082; 1:400) to mark the activated hepatic stellate cells at 4°C overnight. Secondary antibody incubation was performed with Goat anti-Mouse IgG H&L Alexa Fluor® 555 (Thermo; A21422; 2μg/ml) in combination with 4′,6-diamidino-2-phenylindole (DAPI) for 1 h at RT. Conjugated desmin (Thermo; eFluor™ 660; 50-9747-82; 10μg/ml) was used to detect proliferating hepatic stellate cells at 4°C overnight and then in combination with DAPI for 1 h at RT. Activated endothelial cells were marked by von Willebrand factor (vWF) (Thermo; PA5-80223; 2μg/ml) at 4°C overnight. Secondary antibody incubation was performed with Goat anti-Rabbit IgG H&L Alexa Fluor® 488 (Abcam; 150077; 1:500) in combination with DAPI for 1 h at RT. All slides were then mounted with fluorescent aqueous mounting medium (Dako; S302380-2) and dried overnight. Then sections were captured using a 3DHistech Slide scanner, (Panoramic 250 Flash II). The α-SMA stained areas were quantified using Qupath-0.4.3. Desmin and vWF positive stained cell numbers were quantified using Qupath-0.4.3.

Western Blot

Cells on the culture plate were lysed in RIPA Buffer. Protein concentrations were determined using the Pierce™ BCA Protein Assay Kit (Thermo #23225). Protein extracts were boiled in RIPA buffer (Sigma #R0278) and reducing sample buffer (Thermo #39000) at 95 °C for 10 min. A total of 15 µL samples (10ug of protein/lane) were separated by sodium dodecyl sulfate-polyacrylamide gel electrophoresis (SDS-PAGE) using Bolt™ Bis-Tris Plus Mini Protein Gels, 4-12%, 1.0 mm, WedgeWell™ format 12 wells (Thermo #NW04125BOX). Proteins were then transferred onto PVDF membranes, blocked with 5% BSA in TBS-T for 60 min at room temperature, and washed 1 time with TBST. Primary antibody against eNOS polyclonal antibody (Thermo, PA1-037, 1:1000) incubated overnight at 4 °C and 1 hour at RT with HRP-conjugated Goat anti-Rabbit IgG (H+L) Cross-Adsorbed Secondary Antibody (Invitrogen, G21234, 1:1000). Primary antibody against phosphor-eNOS (Ser1177) polyclonal antibody (Thermo, PA5-104858, 1:1000) incubated overnight at 4 °C and 1 hour at RT with HRP-conjugated Goat anti-Rabbit IgG (H+L) Cross-Adsorbed Secondary Antibody (Invitrogen, G21234, 1:1000). Primary antibody against

nitrotyrosine (Abcam, ab42789, 1:250) incubated overnight at 4 °C and 1 hour at RT with HRP-conjugated Goat anti-Rabbit IgG (H+L) Cross-Adsorbed Secondary Antibody (Invitrogen, G21234, 1:1000). HRP conjugated anti-GAPDH antibody (Abcam, #201822, 1:10000) was used for loading control.

In liver tissue (same preparation as described above), a total of 15 µL samples (10ug of protein/lane) were separated by SDS-PAGE using Bolt™ Bis-Tris Plus Mini Protein Gels, 4-12%, 1.0 mm, WedgeWell™ format 12 wells (Thermo #NW04125BOX). Proteins were then transferred onto PVDF membranes. The blotted membranes were immersed in 1X iBind™ Flex Solution (Thermo Scientific #SLF1020) for 10 mins at RT. The immunodetection was done using iBind Flex Western System (Thermo Scientist #SLF1000). The membrane was incubated 2.5 h at RT with primary antibody against α-SMA (Thermo Scientific, #14-9760-82, 1:100), and then with HRP-conjugated Goat Anti-Mouse IgG & IgM Antibody (Merck, AP130P, 1:400). HRP Anti-GAPDH antibody (Abcam, ab201822, 1:10000) as a loading control. All the membrane was incubated with chemiluminescence detection reagents and visualized by the Fusion FX system.

Systemic Inflammation Multiplex Immunoassay

Plasma from cirrhotic rats was collected and analyzed using the ProcartaPlex Immunoassay (Thermo). ProcartaPlex assays are multiplex immunoassays that utilize antibody-coated magnetic beads and are performed in the Bio-Plex 3D Suspension Array system, enabling the simultaneous detection and quantification of multiple cytokines and chemokines. Following hemodynamics measurements, rat blood was collected from the inferior vena cava into EDTA K3E-coated tubes and centrifuged (800 RCF, 20 mins, 4°C) to obtain plasma. Later, Plasma samples were centrifuged at 10,000 × g for 10 minutes to remove particulates. Subsequently, 25 µl of plasma was diluted with 25 µl of Universal Assay Buffer (Thermo). 25 µl of dilution was incubated in a customized ProcartaPlex plate for 2 h with shaking at RT and then washed three times. Subsequently, 25 µl Detection Antibody Mix was added, shaken at RT for 30 mins, and washed three times. Later, add 50 µl of Streptavidin-PE solution, shaking at RT for 30 mins with three times washing. In the end, 120 µl Reading Buffer was added and the plate was shaken at RT for 5 mins. Data was acquired on the Bio-Plex 3D Suspension Array system and analyzed using Bio-Plex Manager Software 6.0.

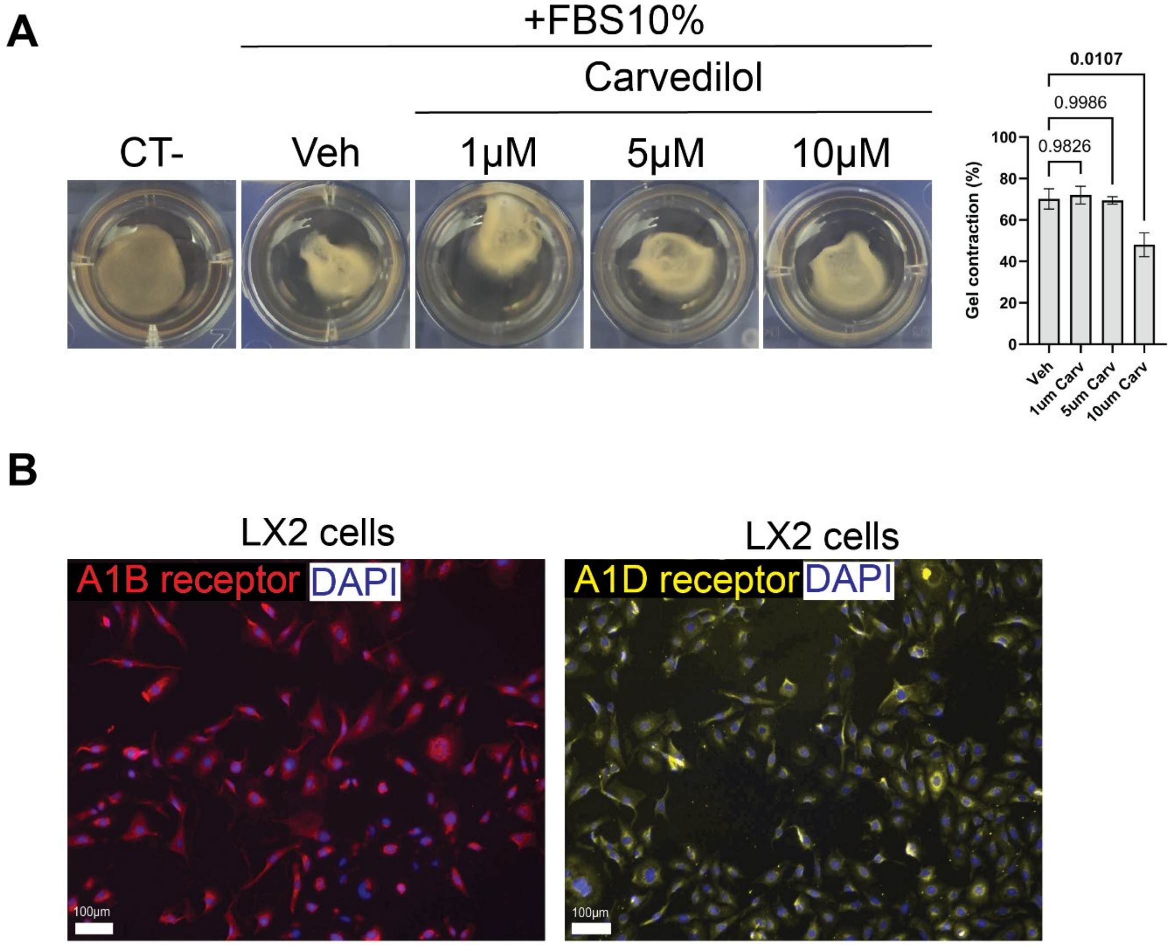

Fig. S1. Carvedilol inhibits human hepatic stellate cells contraction. A) Cell contraction assay in response to different concentrations of Carvedilol in LX2 cells. N=4. Sample distributions were assessed for normality (Kolmogorov−Smirnov test). Normally distributed data were compared to the vehicle with one-way ANOVA (multiple comparison Tukey). All data are represented as mean ± sem. P-value <0.05 was considered significant and depicted in the figure. B) alpha1B-adrenoceptor and alpha1D-adrenoceptor expression verified on LX2 cells. Representative images were captured using the EVOS 5000 system, 20x objective. N=3.

	Vehicle	Propranolol	Carvedilol
**ALT (U/l)**	68.7 ± 5.0	70.2 ± 6.2	62.4 ± 3.1
**AST (U/l)**	89.3 ± 10.5	101.0 ± 10.4	86.4 ± 6.9
**Bilirubin (µmol/l)**	3.1 ± 0.1	3.1 ± 0.2	2.9 ± 0.0
**Creatinine (µmol/l)**	58.9 ± 9.2	42.5 ± 9.4	41.8 ± 2.0
**Total Protein (g/l)**	52.8 ± 0.9	48.9 ± 0.7	48.9 ± 1.7
**Liver weight (gr)**	14.6 ± 1.3	14.0 ± 0.7	14.3 ± 1.3
**Body weight (gr)**	393.4 ± 29.4	389.0 ± 13.9	391.6 ± 26.4
**Spleen weight (gr)**	1.1 ± 0.1	1.2 ± 0.1	1.2 ± 0.1
**n**	8	6	8

Table S1. Blood analysis and corresponding organ weight from 12-week TAA cirrhotic rats treated with vehicle, propranolol or carvedilol. Kruskal−Wallis followed by Dunn's multiple comparisons were performed. All data are represented as mean ± sem. All data are represented as mean ± sem. P-value <0.05 was considered significant. No statistical significance were found.

Comparison of carvedilol effects in advanced and early cirrhosis rats

	Advanced cirrhosis (TAA 12w)			Early cirrhosis (TAA 9w)		
Parameters	Vehicle	Carvedilol	Relative ratio (Carv/Veh)	Vehicle	Carvedilol	Relative ratio (Carv/Veh)
Number of animals	8	8	/	9	10	/
PP (mmHg)	20.0 ± 1.7	15.4 ± 2.8*	- 22%	16.7 ± 1.5	14.0 ± 1.9*	-17%
PBF/BW (ml/min/100gr)	5.1 ± 1.1	4.1 ± 0.8	- 19%	4.8 ± 1.2	4.2 ± 1.0	- 13%
MAP (mmHg)	96 ± 6	84 ± 12	- 12%	94 ± 9	82 ± 7*	- 13%
Heart rate (Bpm)	274 ± 26	249 ± 35	- 9%	296 ± 26	255 ± 20*	- 14%
Liver weight (g)	14.7 ± 3.8	14.3 ± 3.7	- 0.6%	14.0 ± 2.9	14.0 ± 3.6	+ 0.1%
Body weight (g)	393 ± 83	392 ± 75	- 0.4%	369 ± 87	383 ± 95	+ 3%
CPA (%)	19.8 ± 2.9	14.6 ± 2.7*	- 26.8%	17.0 ± 1.9	13.1 ± 3.1*	- 23.1%
α-SMA (%)	3.8 ± 0.5	2.9 ± 0.4*	- 22.7%	3.5 ± 0.5	2.9 ± 0.6*	- 17.4%
Desmin (number)	7 ± 1	5 ± 1*	- 29.8%	6 ± 1	5 ± 1*	- 20.4%
vWF (number)	6 ± 1	5 ± 1*	- 26.3%	5 ± 1	4 ± 1*	- 16.9%
DHE (intensity)	1 ± 0.2	0.7 ± 0.3	- 26.1%	1 ± 0.3	0.5 ± 0.2*	- 52.9%

NOTE. Values represent mean ± standard deviation.

TAA, thioacetamide; PP, portal pressure; PBF, portal blood flow; BW, body weight; MAP, mean arterial pressure; CPA, collagen proportionate area; α-SMA, alpha-smooth muscle actin; vWF, Von Willebrand factor; DHE, dihydroethidium

*P < 0.05 vs vehicle.

Table S2. Comparison of carvedilol effects in advanced and early cirrhosis rats. Sample distributions were assessed for normality (Kolmogorov−Smirnov test). Normally distributed data were compared with unpaired Student t-test otherwise with Mann−Whitney test. All data are represented as mean ± s.d. *P <0.05 was considered significant.

Supplementary reference

1. **Fernández-Iglesias A, Ortega-Ribera M**, Guixé-Muntet S, Gracia-Sancho J. 4 in 1: Antibody-free protocol for isolating the main hepatic cells from healthy and cirrhotic single rat livers. J Cell Mol Med. 2019;23(2):877-86.
