# Supplementary material for: Carvedilol decreases hepatic vascular resistance by reducing fibrogenesis and reversing endothelial dysfunction in cirrhotic rats
Source: JHEP Rep. 2025 Nov 20;8(3):101681. doi: 10.1016/j.jhepr.2025.101681 (PMC12878606; doi:10.1016/j.jhepr.2025.101681)
Supplement: Multimedia component 2 [file mmc2.docx]

**JHEP Reports**

**CTAT methods**

Tables for a “Complete, Transparent, Accurate and Timely account” (CTAT) are now mandatory for all revised submissions. The aim is to enhance the reproducibility of methods.

- Only include the parts relevant to your study
- Refer to the CTAT in the main text as ‘Supplementary CTAT Table’
- Do not add subheadings
- Add as many rows as needed to include all information
- Only include one item per row

**If the CTAT form is not relevant to your study, please outline the reasons why:**

| / |
| --- |

- 1. **Antibodies**

| **Name** | **Citation** | **Supplier** | **Cat no.** | **Clone no.** |
| --- | --- | --- | --- | --- |
| BrdU Monoclonal Antibody |  | Thermo Fisher | MA3-071 | BU-1 |
| Goat anti-Mouse (Alexa Fluor™ 555) |  | Thermo Fisher | A-21422 | Polyclonal |
| Anti-alpha-smooth muscle actin |  | Thermo Fisher | 14-9760-82 | 1A4 |
| Conjugated Desmin Monoclonal Antibody |  | Thermo Fisher | 50-9747-82 | DE-U-10 |
| VWF Polyclonal Antibody |  | Thermo Fisher | PA5-80223 | Polyclonal |
| Goat Anti-Rabbit IgG H&L (Alexa Fluor® 488) |  | abcam | ab150077 | Polyclonal |
| eNOS Polyclonal Antibody |  | Thermo Fisher | PA1-037 | Polyclonal |
| Goat anti-Rabbit Secondary Antibody, HRP |  | Thermo Fisher | G-21234 | Polyclonal |
| Phospho-eNOS (Ser1177) Polyclonal Antibody |  | Thermo Fisher | PA5-104858 | Polyclonal |
| Anti-Nitrotyrosine antibody |  | abcam | ab42789 | Polyclonal |
| HRP Anti-GAPDH antibody |  | abcam | ab201822 | EPR16891 |
| Goat Anti-Mouse Antibody, HRP conjugate |  | Merck | AP130P | Polyclonal |
| alpha-1b Adrenergic Receptor Antibody |  | Thermo Fisher | PA5-114827 | Polyclonal |
| alpha-1D Adrenergic Receptor Antibody |  | Thermo Fisher | PA5-72171 | Polyclonal |

- 1. **Cell lines**

| **Name** | **Citation** | **Supplier** | **Cat no.** | **Passage no.** | **Authentication test method** |
| --- | --- | --- | --- | --- | --- |
| Human Umbilical Vein Endothelial Cells |  | Lonza | C2519A | P0 |  |
| hepatic stellate cell line (LX2) |  | IDIBAPS Biomedical Research Institute |  | P15 |  |

- 1. **Organisms**

| **Name** | **Citation** | **Supplier** | **Strain** | **Sex** | **Age** | **Overall n number** |
| --- | --- | --- | --- | --- | --- | --- |
| Wild Type |  | Inotiv&Envigo | Sprague-Dawley | Male and Female | 12 w to 26 w (TAA period included) | 50 |

- 1. **Sequence based reagents**

| **Name** | **Sequence** | **Supplier** |
| --- | --- | --- |
| Not using |  |  |

- 1. **Biological samples**

| **Description** | **Source** | **Identifier** |
| --- | --- | --- |
| Rat liver tissue | Department of Visceral Surgery and Medicine, Inselspital, Bern University Hospital |  |

- 1. **Deposited data**

| **Name of repository** | **Identifier** | **Link** |
| --- | --- | --- |
|  |  |  |

- 1. **Software**

| **Software name** | **Manufacturer** | **Version** |
| --- | --- | --- |
| ImageJ | NIH and University of Wisconsin | V 1.53f |
| Qupath | University of Edinburgh | 0.4.3 |
| Bio-Plex Manager Software | Bio-Rad | 6.0 |
| LabChart | ADINSTRUMENTS | V5.5.6 |
| GraphPad Prism | Dotmatics | V10.0 |

- 1. **Other (*e.g*. drugs, proteins, vectors etc.)**

| Carvedilol | Hexal AG, Germany |  |
| --- | --- | --- |
| Propranolol | Ratiopharm, Germany |  |
| Methoxamine | Merck: M6524 |  |
| isoflurane | Girovet: 469860 |  |
| Dihydroethidium | Sigma, D7008 |  |
| DAF-FM-DA | ThermoFisher: D23844 |  |
| PPX-14-MXDJZYM  Thermo Fisher | Customized multiplex immunoassays |  |

- 1. **Please provide the details of the corresponding methods author for the manuscript:**

| Jaume Bosch  Department of Visceral Surgery and Medicine, Inselspital, Bern University Hospital, University of Bern  Jaime.bosch@unibe.ch |
| --- |

**2.0 Please confirm for randomised controlled trials all versions of the clinical protocol are included in the submission. These will be published online as supplementary information.**

|  |
| --- |
