# Supplementary material for: Carvedilol decreases hepatic vascular resistance by reducing fibrogenesis and reversing endothelial dysfunction in cirrhotic rats
Source: JHEP Rep. 2025 Nov 20;8(3):101681. doi: 10.1016/j.jhepr.2025.101681 (PMC12878606; doi:10.1016/j.jhepr.2025.101681)
Supplement: Multimedia component 4 [file mmc4.pdf]

Carvedilol decreases hepatic vascular resistance by reducing fibrogenesis and reversing endothelial dysfunction in cirrhotic rats

Yeldos Nulan[1,2,†], **Eric Felli**[1,2,†,*], Sonia-Emilia Selicean[1,2], Manuel Prampolini[1,2], Annalisa Berzigotti[1,2], Jordi Gracia-Sancho[1,2,3], Jaume Bosch[1,2]

JHEP Reports **2026**. vol. 8 | 1–13

Check for updates

Background & Aims: Cirrhosis increases hepatic vascular resistance (IHVR) by disrupting liver architecture due to fibrosis, and by elevating hepatic vascular tone due to hepatic endothelial dysfunction. IHVR increases portal pressure (PP), later aggravated by increased portal blood inflow. Carvedilol, a third-generation non-selective β-blocker with anti-α1-adrenergic activity, reduces PP more than propranolol, likely decreasing IHVR. However, its intrahepatic effects remain largely unexplored. This study aimed to address these issues.

Methods: Human cell lines (LX2 and HUVECs) and primary liver sinusoidal endothelial cells (LSECs) and hepatic stellate cells (HSCs) isolated from cirrhotic rats (12-week thioacetamide [TAA] model) were treated with vehicle, carvedilol (10 μM), or propranolol (10 μM). Nitric oxide release, oxidative stress, and cell contraction were assessed. Cirrhotic rats were treated with vehicle, carvedilol (10 mg/kg/day for 2 weeks), or propranolol (30 mg/kg/day for 2 weeks) at early and advanced stages of cirrhosis (9 and 12 weeks of TAA). Hepatic hemodynamics, liver fibrosis, antioxidant activity, and inflammatory biomarkers were evaluated.

Results: Carvedilol increased nitric oxide release in LSECs and HUVECs and significantly reduced contraction of HSCs and LX2 cells in cirrhotic conditions. *In vivo*, carvedilol significantly reduced PP in both early and advanced cirrhosis (12-week TAA: –22%, $p = 0.0008$; 9-week TAA: –17%, $p = 0.0038$), decreased liver fibrosis area (–26.8%, $p = 0.0013$ *vs.* –23.1%, $p = 0.0047$), and reduced α-SMA expression (–22.7%, $p = 0.0018$ *vs.* –17.4%, $p = 0.0455$). Additionally, carvedilol improved endothelial dysfunction and reduced oxidative stress and inflammation. Propranolol did not exert these beneficial effects and produced a smaller, non-significant reduction in PP (–7%, $p = 0.4029$).

Conclusions: The greater reduction in PP achieved with carvedilol is mediated not only by its non-selective β-blocker effects but, more importantly, by its ability to reverse hepatic endothelial dysfunction, reduce fibrosis, enhance antioxidant activity, and exert moderate anti-inflammatory effects. These findings support extending the use of carvedilol even to patients without overt signs of portal hypertension.

© 2025 The Authors. Published by Elsevier B.V. on behalf of European Association for the Study of the Liver (EASL). This is an open access article under the CC BY license (http://creativecommons.org/licenses/by/4.0/).

Introduction

Cirrhosis is a leading cause of mortality worldwide, ranking 5th among adults.[1,2] The high mortality associated with cirrhosis is due to complications arising from portal hypertension, ascites, variceal bleeding, hepatic encephalopathy, and hepatocellular carcinoma.[3] In cirrhosis, portal hypertension occurs because of increased resistance to blood flow within the portal venous system, and is later aggravated by splanchnic vasodilation and increased portal blood inflow. The increased resistance is caused by structural changes in the liver (fibrosis, tissue remodeling, formation of regenerative nodules, and angiogenesis),[4,5] together with an increased hepatic vascular tone, due to liver sinusoidal endothelial cell (LSEC) dysfunction, characterized by reduced production of vasodilators like nitric oxide (NO)[6] and hypersensitivity to vasoconstrictor stimuli.[7]

Suppression of the cause of cirrhosis is the only therapy that may improve cirrhosis,[8] while anti-fibrotic therapies are under development.[9] Non-selective β-blockers (NSBBs), such as propranolol or nadolol,[10] are the mainstay of therapy for portal hypertension. NSBBs reduce portal pressure by decreasing portal blood inflow by means of lowering cardiac output through β1-adrenoceptor (β1-AR) blockade and by inducing vasoconstriction in the splanchnic circulation via β2-adrenoceptor (β2-AR) blockade. Carvedilol, a third-generation NSBB with intrinsic anti α-1 adrenoceptor activity (α1-AR), is at present the NSBB of choice for portal hypertension because it

* Corresponding authors. Addresses: Department of Visceral Surgery and Medicine, Inselspital, Bern University Hospital, University of Bern; Department for Biomedical Research, University of Bern, Murtenstrasse 35, 3008, Bern, Switzerland.
E-mail address: eric.felli@unibe.ch (E. Felli).
† These authors contributed equally to the manuscript.
https://doi.org/10.1016/j.jhepr.2025.101681

achieves a greater portal pressure reduction compared to propranolol,[11] which is thought to be due to a decrease in the hepatic vascular tone.[12] Studies in cardiovascular biology have shown that carvedilol has pleiotropic effects that go beyond adrenergic blockade, including protection against endothelial dysfunction by increasing NO release, anti-oxidant activity, and decreased vascular remodeling.[13] These effects suggest that, in cirrhosis, carvedilol might deactivate hepatic stellate cells (HSCs), which are key drivers of disease progression, in part by blocking α1-adrenergic signaling.[14] Surprisingly, besides these important connotations, there is scarce knowledge about the intrahepatic effects of carvedilol in cirrhosis, which prompted us to perform the current study.

Materials and methods

Animal models of cirrhosis

Female and male Sprague-Dawley rats were maintained under standard environmental conditions at the University of Bern animal facility. The conditions included a 12-hour light/dark cycle and 10-15 air exchanges per hour. Upon arrival, the rats were acclimatized for 2 weeks before starting any experiments or treatments. Throughout the study, rats had free access to chow diet and water. All experimental procedures were approved by the Bern Cantonal Ethics Committee and adhered to the Laboratory Animal Care guidelines (approval number BE382022). Cirrhosis and portal hypertension was induced by the twice weekly administration of thioacetamide (TAA) (Sigma-Aldrich; Ref. No.172502) (200 mg/kg of body weight; intraperitoneally) for a total of 12 weeks. In addition, other groups of animals received TAA for only 9 weeks to model early cirrhosis. Before each TAA administration, the rats' body weights were recorded, and the TAA dosage was adjusted accordingly.

Isolation of liver endothelial sinusoidal cells and hepatic stellate cells

The isolation of primary liver cells was conducted following methods previously described.[15] Further details are given in supplementary methods.

Cell culture

Freshly isolated LSECs were cultured in RPMI 1640 Medium with 10% FBS, 1% antibiotic-antimycotic, 50 μg/ml endothelial cell growth supplement, and 100 μg/ml heparin. HSCs were maintained in Iscove's modified Dulbecco's medium with 10% FBS and 1% antibiotic-antimycotic. HUVECs (P2–P4, C2519A, Lonza) were cultured in Medium 199 with 20% FBS, 1% antibiotic-antimycotic, 50 μg/ml endothelial cell growth supplement, and 100 μg/ml heparin. LX-2 cells (P15–P20, Liver Vascular Biology Research Group, IDIBAPS Biomedical Research Institute) were cultured in Dulbecco's modified eagle medium/nutrient mixture F-12 with 10% FBS and 1% antibiotic-antimycotic. All cells were incubated at 37 °C in a humid atmosphere (95%) with 5% CO_2. Please refer to the supplementary methods.

Measurement of intracellular nitric oxide levels

Intracellular nitric oxide levels in LSECs and HUVECs were assessed with 4-amino-5-methylamino-2', 7'-dichlorofluorescein diacetate (DAF-FM-DA, ThermoFisher: D23844). LSECs and HUVECs were treated with carvedilol or vehicle for 24 h, and HUVECs with propranolol or vehicle for 24 h. After, cells were loaded with DAF-FM-DA (10 μM) and nucleus biomarker Hoechst (ThermoFisher: 33342) in the incubator for 30 mins. Cells were washed to remove excess probe, replaced with fresh medium, and then incubated for an additional 15 mins period to allow complete de-esterification of the intracellular diacetate. Then, cells were rinsed three times with PBS (ThermoFisher: 10010023), protected from light, and placed on an inverted fluorescence microscope (LEICA-DMI4000). Fluorescence images were obtained by setting the 488 nm (excitation) and 505 to 530 nm (emission) filters for DAF-FM-DA with a 10x or 20x objective.

Cell contraction

Cell contraction assays were conducted using HSCs and LX2 cells in collagen lattices. Freshly isolated HSCs were cultured for 5 days to reach full activation. Collagen gels (1.3 mg/ml) containing HSCs or LX2 cells (1.5×10^5 cells/ml) were prepared in 24-well plates pre-treated with 1% BSA. After gel solidification, serum-free media with 10 μM carvedilol, propranolol or vehicle was added for 24 h. Contraction was induced with 10% FBS for 48 h, with photographs taken at 1, 24, and 48 h. In a separate experiment, 500 μM methoxamine was used to foster α1-adrenergic contraction, and the effects of carvedilol and propranolol were assessed at 1 h. Please see details in supplementary methods.

Cell proliferation assay

The proliferation of LX2 cells was assessed using a bromodeoxyuridine (BrdU) incorporation assay. Cells were cultured in 6-well plates for 24 h, treated with or without carvedilol (10 μM) for 24 h, and labeled with BrdU for 2 h. Following fixation with 4% PFA, cells were permeabilized with 0.1% Triton X-100, treated with hydrochloric acid, neutralized with phosphate/citric acid buffer, and washed. BrdU was detected using a primary antibody (Thermo: MA3-071, 1:100) overnight at 4 °C, followed by a fluorescent secondary antibody (Thermo: A21422, 2 μg/ml) for 1 h. Imaging was performed on EVOS 5000 using the RFP channel. Please see details in supplementary methods.

Hepatic and systemic hemodynamics

Cirrhotic rats were administered either carvedilol, 10 mg/kg/day (Hexal AG, Germany); propranolol, 30 mg/kg/day (Ratiopharm, Germany), or vehicle by oral gavage for 2 weeks. One hour after the final dose, the rats were anesthetized with inhaled isoflurane (Girovet: 469860). Mean arterial pressure was measured by cannulating the femoral artery, and portal pressure was measured by cannulating the mesenteric vein, both using a heparinized P50 catheter (Portex) connected to a pressure transducer. Portal blood flow was determined using a non-constrictive perivascular ultrasonic transit-time flow probe (Transonic Systems Inc.) placed close to the liver hilus. Pressure and flow probes were connected to a PowerLab (4SP) data recording system, and data was displayed using LabChart v5.5.6 software. Hemodynamic parameters were recorded following a 30-minute stabilization period, collecting

stable data for 2 minutes. After the hemodynamic study, blood samples were collected. Finally, the animals were euthanized with a pentobarbital overdose (200 mg/kg, Nembutal), and tissue samples were harvested for molecular and histological experiments.

Systemic inflammation multiplex immunoassay

Plasma from cirrhotic rats was collected after hemodynamic measurements, processed via centrifugation, and analyzed using the ProcartaPlex Immunoassay on the Bio-Plex 3D Suspension Array system. The assay involved incubating 25 µl plasma on a customized ProcartaPlex plate, followed by sequential addition and washing of Detection Antibody Mix, Streptavidin-PE, and Reading Buffer, with shaking at room temperature. Data was acquired and analyzed using Bio-Plex Manager Software 6.0. Details are shown in the supplementary methods.

Superoxide detection

Superoxide (O_2^-) levels were measured using the fluorescent dye dihydroethidium (DHE) (10 µM) in liver-frozen sections, HUVECs, and LX2 cells. Liver tissue: Frozen sections (10 µm) were prepared using a Leica Cryostat, loaded with DHE, and incubated at room temperature for 30 minutes. Slides were imaged with fluorescent mounting medium. Cell lines: Cells were rinsed with PBS, incubated with DHE for 30 minutes, washed with PBS, and imaged immediately. Fluorescent images were captured using an EVOS 5000 microscope (10x objective), and the signal was quantified with ImageJ software (v1.53). Please see details in supplementary methods.

Liver histology

Liver tissue was fixed in 4% formaldehyde (Sigma), embedded in paraffin, and sectioned. To evaluate fibrosis, the sections were stained with 0.1% Sirius red in an aqueous picric acid solution (Sigma). Then sections were captured using a 3DHistech Slide scanner (Panoramic 250 Flash II). The Sirius red-stained areas were quantified using Qupath-0.4.3, and the results were expressed as collagen proportionate area (CPA), the percentage of the total tissue section area positively stained for fibrosis (red). Septa were classified as thin *vs*. thick, and incomplete *vs*. complete.

Immunostaining

Liver tissue was fixed in 4% formaldehyde, paraffin-embedded, sectioned, and dewaxed. Antigen retrieval was performed using Heated EDTA-Tris buffer (pH = 9), followed by blocking with 3% goat serum. Primary antibodies were applied overnight at 4 °C to target alpha smooth muscle actin (α-SMA) (1:400, Thermo) for activated HSCs, desmin (10 µg/ml, Thermo) for proliferating HSCs, and von Willebrand factor (vWF) (2 µg/ml, Thermo) for endothelial cells. Secondary antibodies conjugated to Alexa Fluor dyes were incubated with DAPI for 1 h at room temperature. Sections were mounted with fluorescent medium and scanned using a 3DHistech Slide scanner. Quantification of α-SMA–positive stained area and of desmin- and vWF-positive cells was performed using QuPath v0.4.3. Please see supplementary methods for further details.

Western blot

Cells were lysed in RIPA buffer, and protein concentrations measured using the Pierce™ BCA Kit. Samples (10 µg protein/lane) were separated by SDS-PAGE using Bolt™ Bis-Tris Gels and transferred to PVDF membranes. Membranes were blocked, probed with primary antibodies (endothelial nitric oxide synthase [eNOS], phospho-eNOS, nitrotyrosine, α-SMA, GAPDH), and incubated with HRP-conjugated secondary antibodies. Detection was performed with chemiluminescence and visualized using the Fusion FX system. Liver tissue proteins were similarly processed using the iBind™ Flex Western System. GAPDH served as the loading control. Please see supplementary methods for additional details.

Statistical analysis

Statistical analyses were performed using GraphPad Prism 10 (GraphPad Software). Data represent biological replicates (n), including TAA 12 weeks group (vehicle: n = 8; propranolol: n = 6; carvedilol: n = 8) and TAA 9 weeks group (vehicle: n = 9; carvedilol: n = 10), and are depicted in figures as mean values ± standard error. The frequency distribution of data was assessed using a normality test (Kolmogorov–Smirnov). For samples with a normal distribution, means were compared by Student's *t* test (two samples) or ANOVA (more than two samples) followed by Tukey's *post hoc* analysis. Otherwise, non-parametric tests (Kruskal–Wallis) followed by Dunn's multiple comparisons were used. Differences were considered statistically significant at $p < 0.05$.

Results

Carvedilol increases the release of nitric oxide in LSECs

The effect of carvedilol on NO release was studied in HUVECs using the DAF-FM assay (Fig. 1A). In addition to enhancing NO release, HUVECs treated with carvedilol exhibited increased expression of endothelial nitric oxide synthase (eNOS) and its phosphorylated form (phospho-eNOS) (Fig. 1B). Moreover, decreased NO scavenging by O_2^- further increased the availability of NO. Measuring O_2^- levels by DHE showed that carvedilol had antioxidant activity (Fig. 1C). The reduction in superoxide was associated with decreased peroxynitrite ($ONOO^-$) formation, indirectly tested by diminished nitrotyrosinated protein (Fig. 1D). The upregulation of NO availability by carvedilol was further confirmed in freshly isolated LSECs from cirrhotic rats (Fig. 1E).

Carvedilol inhibits HSC contraction via alpha1-blocking activity

A functional cell contraction assay was used to evaluate the response of activated cirrhotic HSCs and LX2 cells to carvedilol (Fig. 2A). Both cell types exhibited a significant reduction in contractility when treated with 10 µM carvedilol (Fig. 2B,C and Fig. S1A). The α1-adrenoceptor expression was verified in LX2 cells (Fig. S1B). The α1-receptor blocking activity of carvedilol was assessed by stimulating LX2 cells with 500 µM of the α1-adrenergic receptor agonist methoxamine (Fig. 2D), where carvedilol significantly inhibited LX2 contraction. In addition, carvedilol showed antioxidant activity in LX2 cells

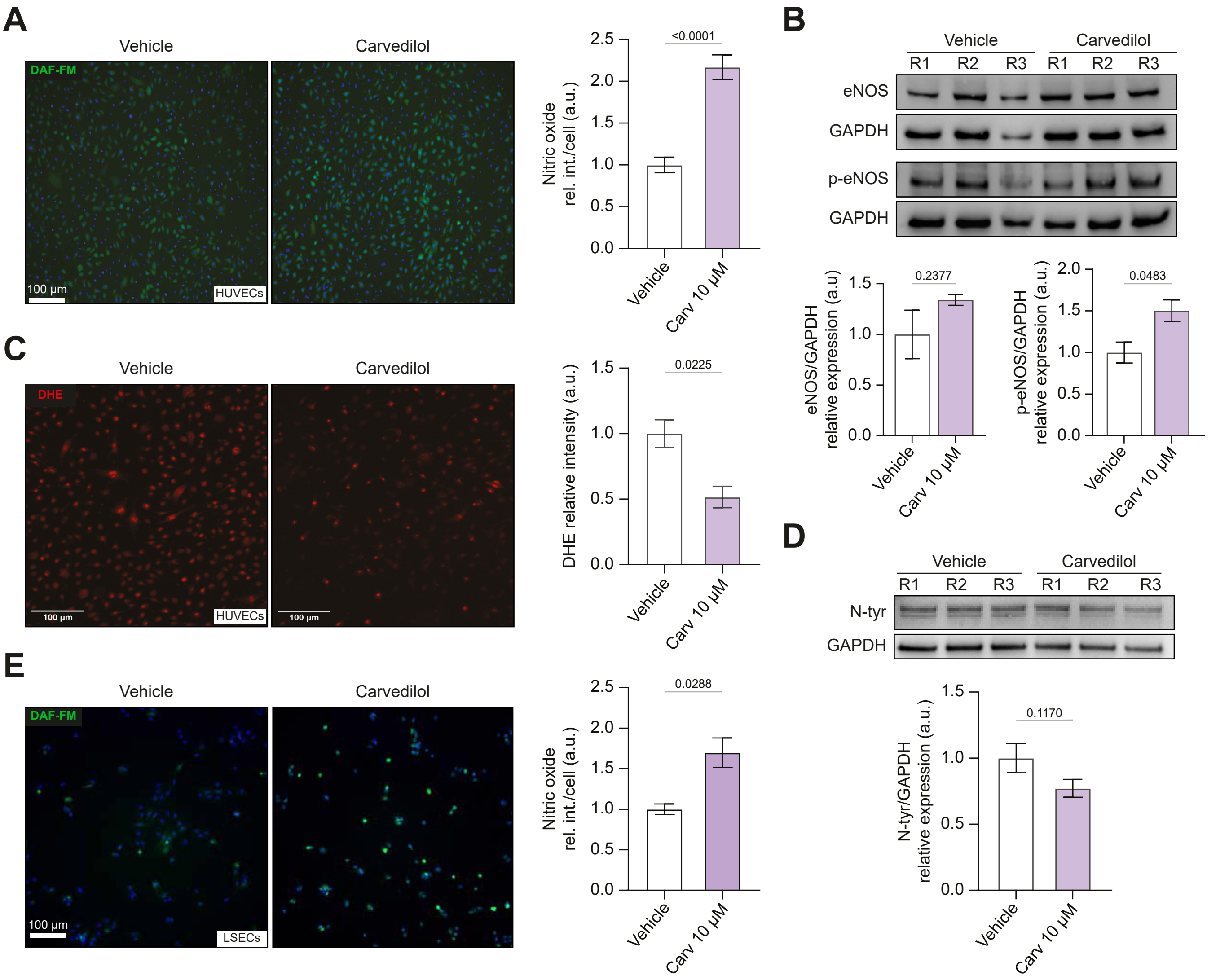

Fig. 1. Carvedilol increases NO release in HUVECs and liver sinusoidal endothelial cells. (A) NO detection using DAF-FM. n = 6. (B) Western blot and the relative semi-quantification of eNOS and p-eNOS from HUVECs treated with and without carvedilol for 24 hours. n = 3. (C) Superoxide levels in HUVECs treated with carvedilol or vehicle. Representative pictures made with EVOS 5000 of *in situ* detection of O_2^- levels with DHE, n = 3. (D) Representative picture of western blot with relative semiquantitative analysis of nitrotyrosinated protein levels measured in HUVECs treated with carvedilol or vehicle. n = 5. (E) NO levels in primary liver sinusoidal endothelial cells isolated from cirrhotic rats incubated for 24 hours with carvedilol or vehicle. n = 4. DAF-FM live-cell imaging was performed with EVOS 5000 and Leica DMI4000B multipurpose fluorescence system. For each experiment, sample distributions were assessed for normality (Kolmogorov–Smirnov test). Normally distributed data were compared using an unpaired Student's *t* test, while non-normally distributed data were compared using the Mann–Whitney test. All data are represented as mean ± SEM. *p* values <0.05 were considered significant and are depicted in the figure. DAF-FM-DA, 4-amino-5-methylamino-2',7'-dichlorofluorescein diacetate; DHE, dihydroethidium; eNOS, endothelial nitric oxide synthase; HUVECs, human umbilical vein endothelial cells; NO, nitric oxide; O_2^-, superoxide.

(Fig. 2E). Cell proliferation, assessed by BrdU incorporation in LX2 cells, was significantly decreased by carvedilol (Fig. 2F).

Effects of carvedilol *vs.* propranolol in endothelial cells and HSCs *in vitro*

A comparative *in vitro* study was conducted to evaluate the effects of carvedilol and propranolol on HUVECs and LX2 cells. Treatment with 10 µM carvedilol increased NO production in HUVECs, while 10 µM propranolol showed only a small non-significant increase (Fig. 3A). To confirm these findings, eNOS expression was assessed; carvedilol upregulated eNOS expression in HUVECs, whereas propranolol did not (Fig. 3B). In LX2 cells, carvedilol, but not propranolol, inhibited cell contraction (Fig. 3C). To confirm that inhibition of cell contraction involves α1-receptor blocking activity, LX2 cells were stimulated with 500 µM methoxamine for 1 h. Carvedilol inhibited contraction in response to methoxamine, but propranolol did not (Fig. 3D). Finally, unlike propranolol, carvedilol decreased superoxide levels in LX2 cells (Fig. 3E).

Carvedilol reduces portal hypertension more effectively than propranolol

The *in vivo* effects of 2-week carvedilol *vs.* propranolol treatment were analyzed in rats with advanced cirrhosis, induced

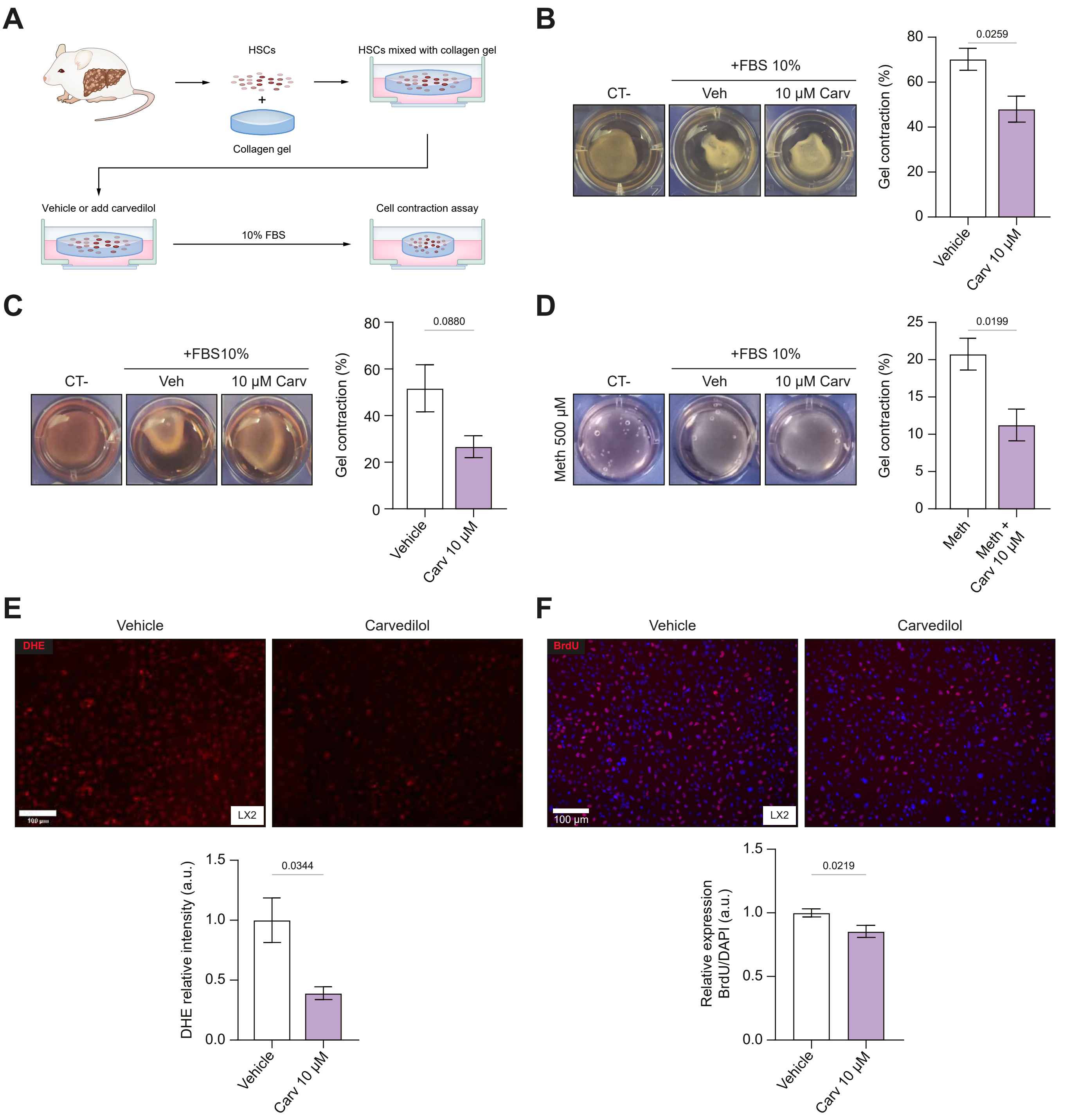

Fig. 2. Carvedilol inhibition of hepatic stellate cell contraction via alpha1 blocking activity. (A) Cell contraction assay illustration, cell number is consistent in each gel (1.5x10^5 cells/ml). (B) Cell contraction assay in LX2. Picture sampled after 48 hours. n = 4. (C) Cell contraction assay in primary HSCs isolated from TAA-cirrhotic rat liver and cultured for 24 hours with carvedilol or vehicle. n = 3. (D) The specific role of α1-receptor blocking activity of carvedilol was assessed by pre-stimulating LX2 cells with α1-adrenoceptor α1-adrenergic receptor agonist methoxamine for 1 h. n = 4. (E) Superoxide levels in LX2 cells treated with carvedilol (cell number = 1,496) or vehicle (cell number = 1,538). Representative pictures made with EVOS 5000 of *in situ* detection of O_2^- with DHE. n = 3. (F) Effects of carvedilol on cell proliferation (BrdU incorporation assay) in LX2 cells. Representative pictures made with EVOS 5000. n = 9. For each experiment, sample distributions were assessed for normality (Kolmogorov–Smirnov test). Normally distributed data were compared using an unpaired Student's *t* test, while non-normally distributed data were compared using the Mann–Whitney test. All data are represented as mean ± SEM. *p* values <0.05 were considered significant and are depicted in the figure. DHE, dihydroethidium; HSCs, hepatic stellate cells; O_2^-, superoxide; TAA, thioacetamide.

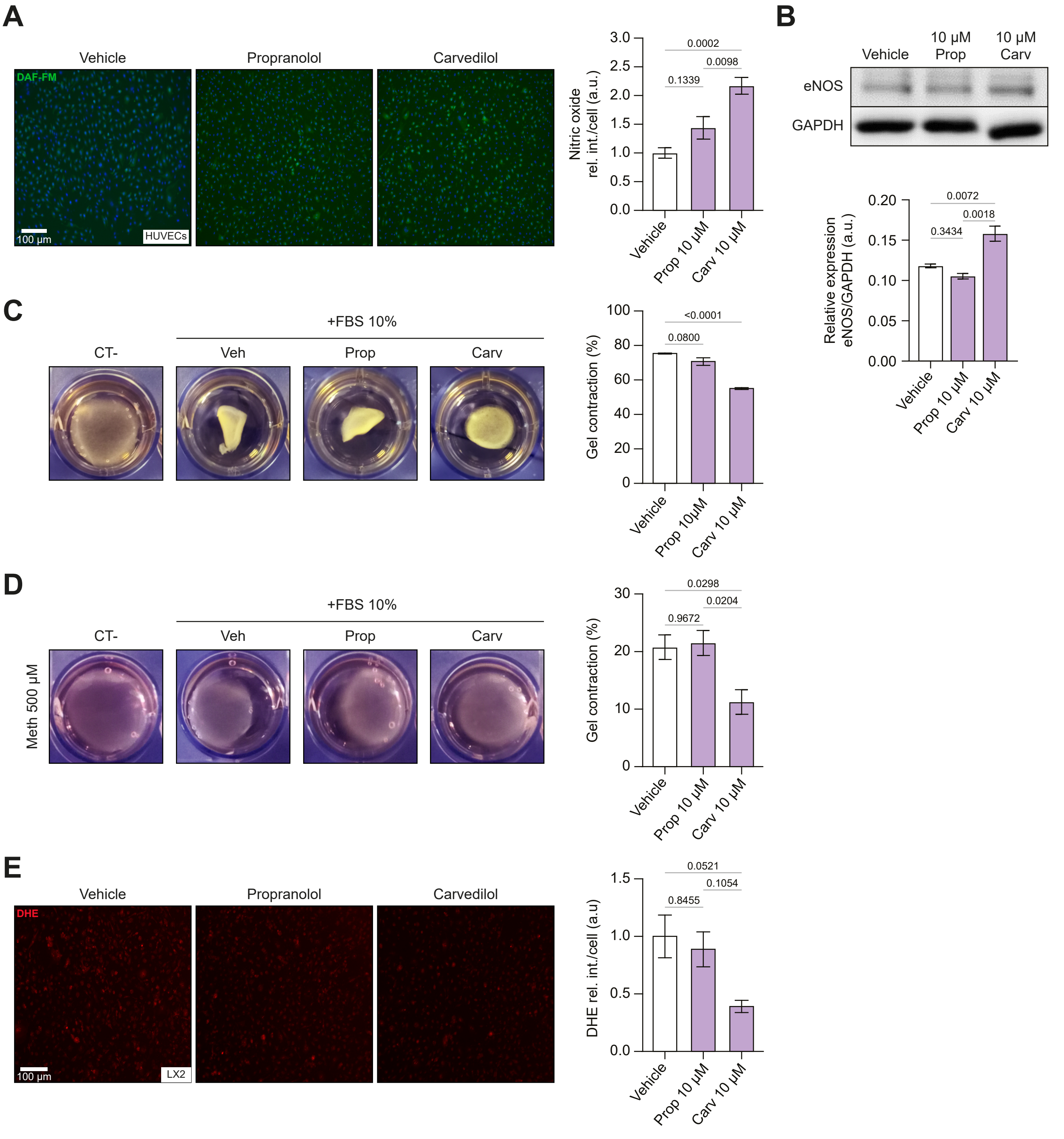

Fig. 3. Comparison of the effects of carvedilol and propranolol *in vitro*. (A) NO production in HUVECs by DAF-FM assay with carvedilol or propranolol. Representative images were captured using the EVOS 5000 system. n = 6. (B) Western blot and relative semi-quantification of eNOS from HUVECs treated with carvedilol or propranolol for 24 h. n = 3. (C) Cell contraction assay with LX2. Picture sampled after 48 h. n = 3. (D) LX2 cells treated for 1 h with and without FBS10% plus α1 adrenergic stimulator methoxamine and vehicle, carvedilol, or propranolol. n = 4. (E) Effect of carvedilol on O_2^- levels in LX2 cells treated with and without carvedilol or propranolol. Representative pictures made with EVOS 5000 of *in situ* detection of O_2^- with DHE. n = 3. For each experiment, sample distributions were assessed for normality (Kolmogorov–Smirnov test). Normally distributed data were compared to vehicle with one-way ANOVA (multiple comparison Tukey), while non-normally distributed data were compared using the Kruskal–Wallis test. All data are represented as mean ± SEM. *p* values <0.05 were considered significant and are depicted in the figure. DAF-FM-DA, 4-amino-5-methylamino-2',7'-dichlorofluorescein diacetate; DHE, dihydroethidium; eNOS, endothelial nitric oxide synthase; HUVECs, human umbilical vein endothelial cells; NO, nitric oxide; O_2^-, superoxide.

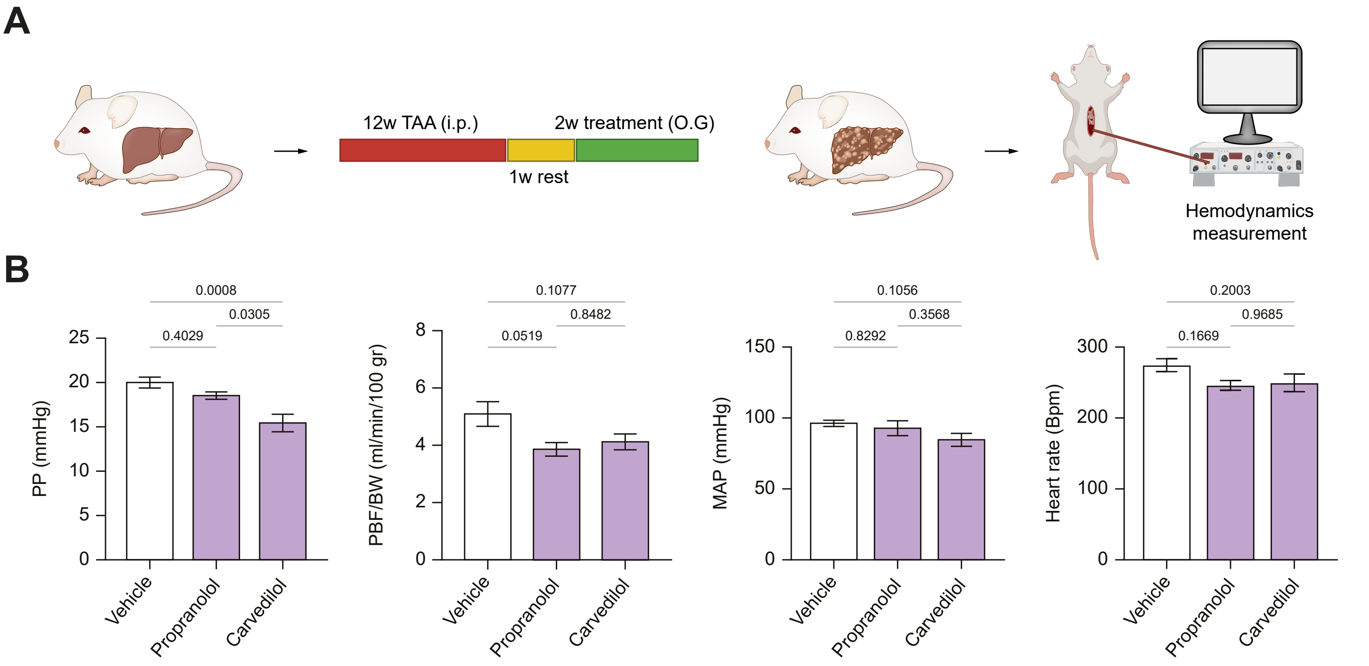

Fig. 4. Hemodynamic effects of carvedilol and propranolol in experimental cirrhosis. (A) Schematic illustration of the cirrhosis induction program and protocol of the experiments. The animals were treated for 12 weeks with TAA, followed by 1-week rest and 2-week oral gavage with drug or vehicle. (B) Effects of the administration of vehicle (n = 8), propranolol (30 mg/kg/day, n = 6), and carvedilol (10 mg/kg/day, n = 8) for 2 weeks on hemodynamic parameters in cirrhotic rats. Sample distributions were assessed for normality (Kolmogorov–Smirnov test). Normally distributed data were compared to the vehicle with one-way ANOVA (multiple comparison Tukey), while non-normally distributed data were compared using the Kruskal–Wallis test. All data are represented as mean ± SEM. p values <0.05 were considered significant and are depicted in the figure. BW, body weight; MAP, mean arterial pressure; PBF, portal blood flow; PP, portal pressure; TAA, thioacetamide.

by 12-week TAA (Fig. 4A). We used only the advanced cirrhosis model in this experiment since the effects of propranolol are less marked in early cirrhosis.[16] Hemodynamic assessments revealed that carvedilol reduced portal pressure significantly more than vehicle and propranolol (Fig. 4B). Carvedilol and propranolol similarly decreased portal blood flow, implying that the greater fall in portal pressure achieved with carvedilol was due to decreased hepatic vascular resistance. Mean arterial pressure was not significantly modified by carvedilol or propranolol, and both similarly reduced heart rate. Neither drug altered kidney function (Table S1). Carvedilol administration favorably influenced liver function, with decreased alanine and aspartate aminotransferase, and a reduction in bilirubin (Table S1).

Carvedilol, but not propranolol, ameliorates hepatic endothelial dysfunction, reduces liver fibrosis, and promotes intrahepatic remodeling in rats with advanced cirrhosis

After hemodynamics, the liver was collected for further analysis. Carvedilol improved endothelial dysfunction, as indicated by a decreased expression of vWF (Fig. 5A) and reduced oxidative stress (DHE staining) in liver tissue sections (Fig. 5B). Carvedilol reduced collagen deposition, as shown in Sirius red-stained sections. Histologically, thinning and occasional disruption of fibrous septa were noted, which suggests architectural improvement, and partial cirrhosis regression[17](Fig. 5C). The percentage of liver tissue occupied by collagen (CPA) decreased by 26% (Table S2). This was likely related to HSC deactivation and decreased proliferation, evidenced by decreased expression of α-SMA and desmin, respectively (Fig. 5D-F). In contrast, propranolol lacks these intrahepatic effects.

Carvedilol consistently reduces portal hypertension and ameliorates liver structure and function in rats with early cirrhosis

To investigate whether the portal pressure reducing effect of carvedilol is consistent across various stages of cirrhosis we tested the effects of a 2-week carvedilol treatment in rats with 9-week TAA intoxication (Fig. 6A). These animals had less structural liver abnormalities, lower CPA, and lower portal pressure than rats receiving TAA for 12-weeks (Table S2). Carvedilol significantly reduced portal pressure, heart rate, and mean arterial pressure compared to vehicle (Fig. 6B). A comparison of carvedilol's effects in 9-week and 12-week cirrhotic rats is summarized in Table S2. Notably, the mean decrease in portal pressure was similar in both cirrhosis stages (advanced cirrhosis -22% *vs.* early cirrhosis -17%, p = 0.3621).

Analysis in liver tissue in 9-week cirrhotic rats showed that carvedilol reduced the number of vWF-positive cells (Fig. 7A) and oxidative stress (Fig. 7B). Histologically, carvedilol improved the altered liver architecture and liver fibrosis by decreasing collagen deposition, as evidenced in Sirius red-stained sections, and by thinning of fibrous septa, which were frequently incomplete (Fig. 7C). This was accompanied by a significant reduction in the CPA (-23%), and by the deactivation and reduced proliferation of HSCs, as evidenced by reduced expression of α-SMA and desmin (Fig. 7D-F).

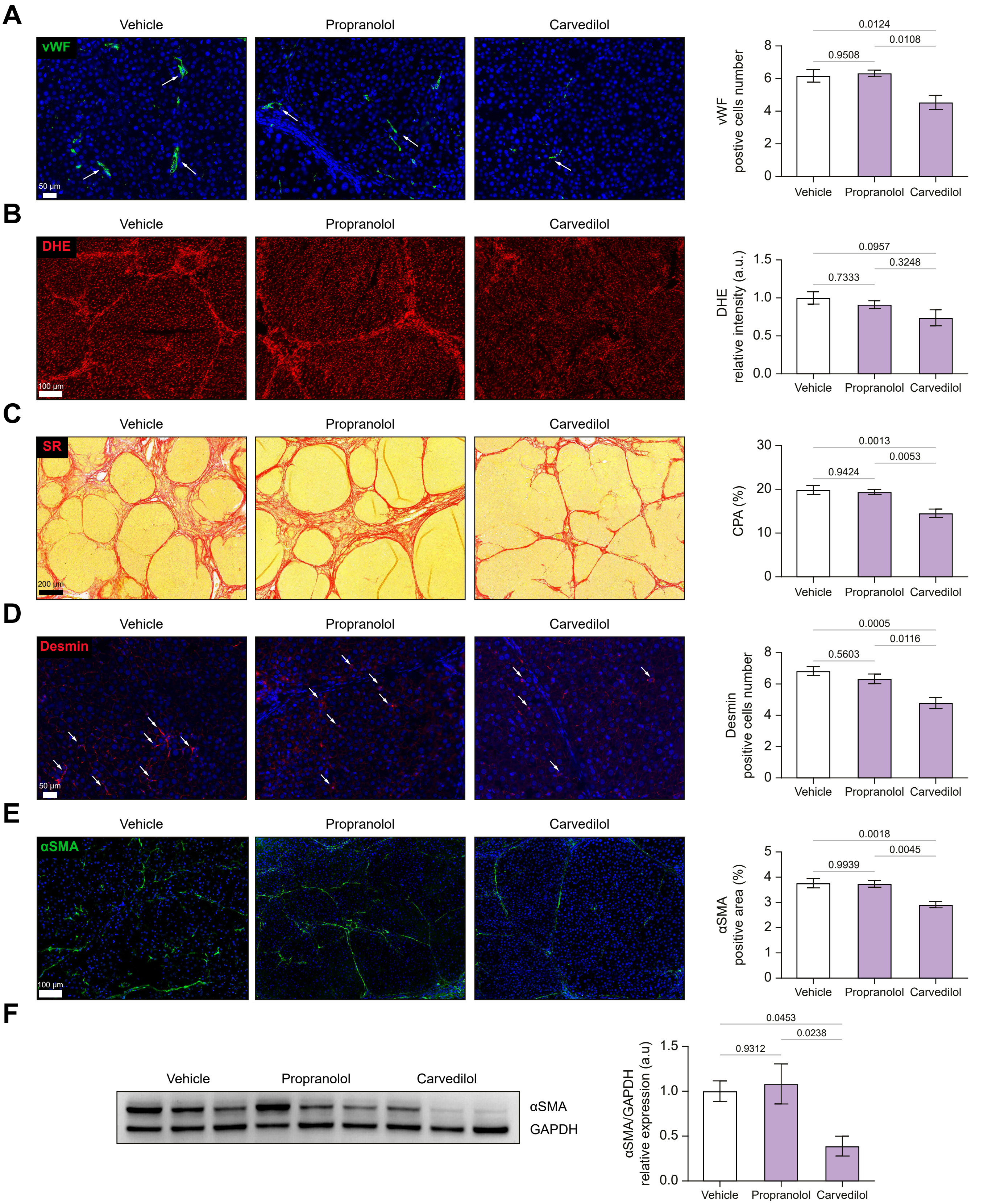

Fig. 5. Effects of carvedilol and propranolol on intrahepatic remodeling in rats with advanced cirrhosis. (A) Endothelial activation assessed as vWF-positive cells. (B) Oxidative stress (O_2^-) was determined in fresh cirrhotic rat liver with dihydroethidium staining. (C–F) Histological and immunostaining evaluation of the liver structural abnormalities was done using Sirius red-stained sections for collagen, αSMA and desmin expression by immunohistochemistry, confirmed by western blot analysis of αSMA and its relative semi-quantification from bulk tissue. Vehicle n = 8; propranolol n = 6; carvedilol n = 8. All images for immunostaining were

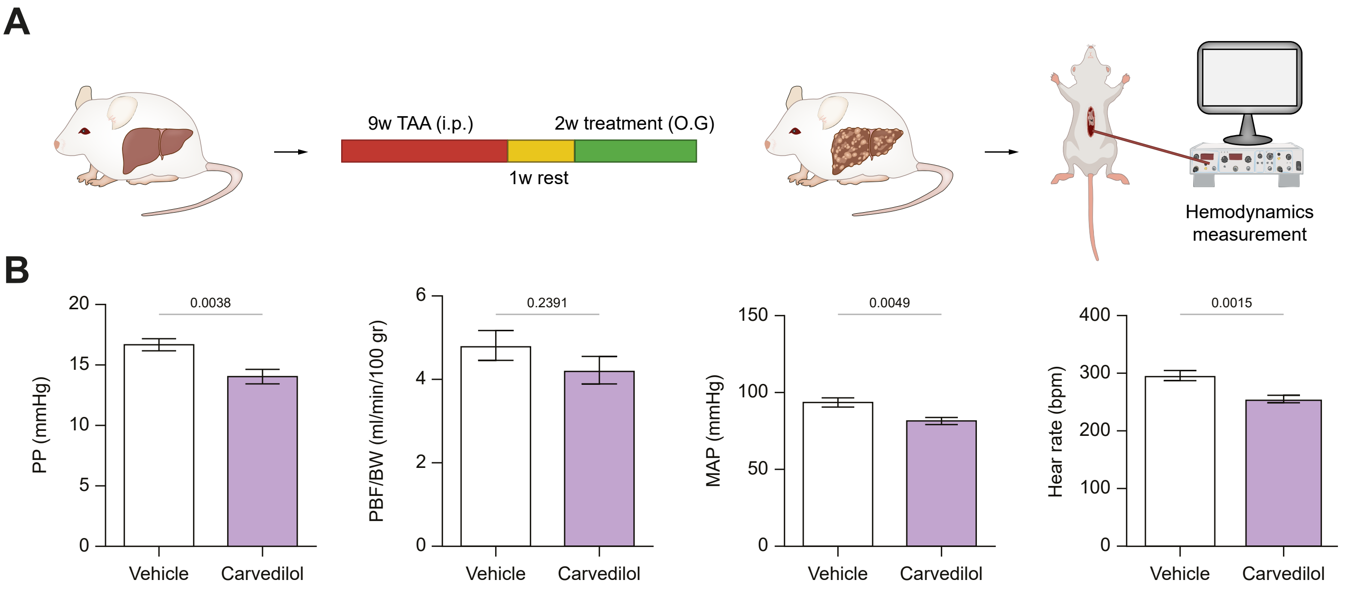

Fig. 6. Hemodynamic effects of carvedilol in rats with early cirrhosis. A) Schematic illustration of cirrhosis induction program and protocol for hemodynamic procedures. (B) Hemodynamic parameters in rats with early cirrhosis treated with vehicle (n = 9) or carvedilol (10 mg/kg/day, n = 10). Sample distributions were assessed for normality (Kolmogorov–Smirnov test). Normally distributed data were compared using an unpaired Student's *t* test, while non-normally distributed data were compared using the Mann–Whitney test. All data are represented as mean ± SEM. *p* values <0.05 were considered significant and are depicted in the figure.

Carvedilol shows anti-inflammatory effects in rats with advanced and early cirrhosis

Chronic inflammation is thought to be a key driver of the progression of cirrhosis. Carvedilol has shown anti-inflammatory effects in various organs.[18–20] The potential anti-inflammatory effects of carvedilol in cirrhosis were evaluated by measuring plasma cytokines (Fig. 8A) and chemokines (Fig. 8B) in rats with advanced cirrhosis (12-week TAA) and early cirrhosis (9-week TAA). Carvedilol non-significantly decreased the pro-inflammatory cytokine tumor necrosis factor (TNF-α) in both stages and increased the anti-fibrotic cytokine interferon-gamma (IFN-γ) in the advanced stage. With regards to chemokines, carvedilol significantly reduced C-X-C motif chemokine ligand 10 (CXCL10) in early cirrhosis, but not in the advanced stage. Moreover, chemokine ligand 5 (CCL5) was decreased by carvedilol in both stages.

Discussion

NSBBs have been the mainstream drug therapy in the management of portal hypertension for decades.[21] However, the third-generation NSBB, carvedilol, has become the preferred treatment for portal hypertension[22,23] after studies demonstrated that it reduces portal pressure to a larger extent and has greater clinical efficacy compared to traditional NSBBs like propranolol and nadolol.[24] Moreover, carvedilol has been shown to prevent decompensation and increase survival of patients with compensated cirrhosis in an individual patient data meta-analysis of different studies.[25] This improvement in prognosis suggests that besides its effects of lowering portal pressure, carvedilol may favorably influence the underlying liver disease. However, there are few studies on its biological effects on cirrhosis, even if some early investigations reported decreased liver fibrosis in carbon tetrachloride-treated rats.[26] Also, although the additional reduction in portal pressure achieved with carvedilol has been attributed to decreased liver vascular resistance,[27] no study so far adequately assessed its mechanism(s). The current study aimed to clarify these issues.

Our study demonstrated that carvedilol exerts beneficial effects on LSEC and HSC pathobiology within the cirrhotic liver. Indeed, carvedilol markedly increased NO bioavailability, increased eNOS expression and preserved eNOS phosphorylation, indicating improved LSEC functionality. Moreover, NO availability was further increased due to reduced scavenging of NO by free oxygen radicals to form peroxynitrite ($ONOO^-$), which has deleterious effects by causing protein nitrotyrosination.[28] Indeed, carvedilol had marked antioxidant activity, as evidenced by reduced O_2^- in HUVEC and LX2 cells, and in fresh-frozen cirrhotic liver slides.[5] Notably, carvedilol markedly inhibited HSC contractility, which was not seen after equimolar doses of propranolol. These differential effects are partially due to the presence of a carbazole moiety in the carvedilol molecule, which leads to strong antioxidant activity.[29] The mechanism by which carvedilol enhances NOS expression and phosphorylation is incompletely understood, but it has been proposed to involve an enhanced expression of β-arrestin,[30] an

sampled with a 3DHistech scanner Panoramic 250 Flash II. For each experiment, sample distributions were assessed for normality (Kolmogorov–Smirnov test). Normally distributed data were compared to the vehicle with one-way ANOVA (multiple comparison Tukey), while non-normally distributed data were compared using the Kruskal–Wallis test. All data are represented as mean ± SEM. *p* values <0.05 were considered significant and are depicted in the figure. O_2^-, superoxide; vWF, von Willebrand factor.

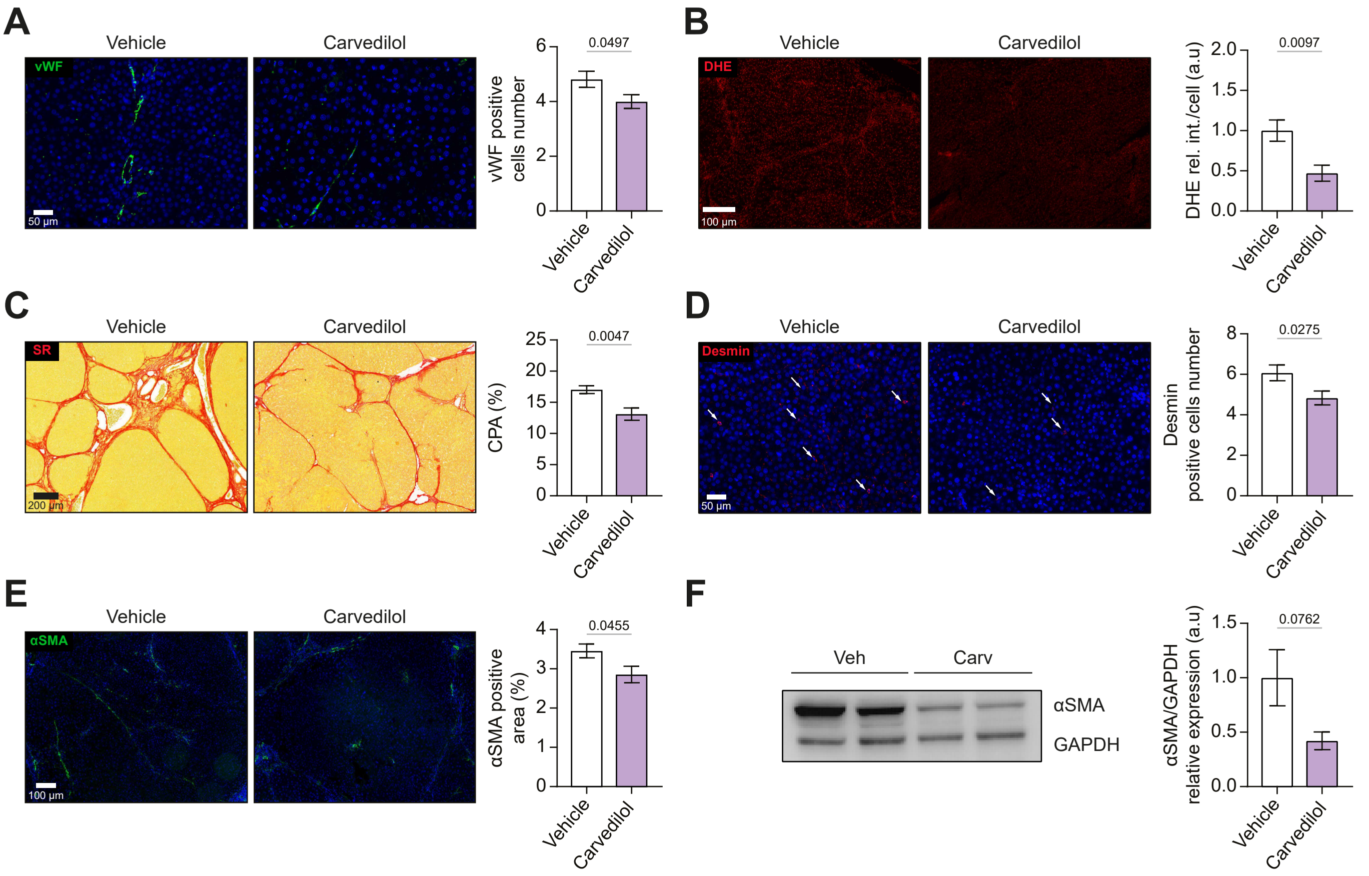

Fig. 7. Effects of carvedilol on intrahepatic remodeling in early cirrhotic rats. (A) Endothelial activation assessed as vWF positive cells. (B) Oxidative stress (O_2^-) determined in fresh liver slices with dihydroethidium staining. (C–F) Histological and immunostaining analyses to assess structural components of cirrhosis in rat liver tissue using Sirius red staining for collagen, and immunostaining for desmin and αSMA, accompanied by a representative Western blot of αSMA with its relative semi-quantification from bulk tissue (vehicle n = 9; carvedilol n = 10). All immunostaining images were captured using a 3DHistech Panoramic 250 Flash II scanner. Sample distributions were assessed for normality (Kolmogorov–Smirnov test). Normally distributed data were compared to the vehicle with one-way ANOVA (multiple comparison Tukey), while non-normally distributed data were compared using the Kruskal–Wallis test. All data are represented as mean ± SEM. *P* values <0.05 were considered significant and are depicted in the figure. O_2^-, superoxide; vWF, von Willebrand factor.

adaptor protein important for the regulation of G-protein-coupled receptors, a property absent in propranolol.[31] However, recent studies showed that the effects of carvedilol can be observed independently of β-arrestin modulation.[32,33] Finally, we showed that inhibition of HSC contraction by carvedilol is partly due to anti-α1-adrenergic blockade.

Together with an increased portal inflow and hepatic vascular tone, portal hypertension is caused by the architectural distortion of the intrahepatic circulation resulting from collagen deposition, fibrosis, vascular remodeling, and formation of regenerative nodules.[6] A major finding of the present study is that carvedilol, but not propranolol, significantly decreases liver fibrosis, as evidenced by reduced CPA and thinning of fibrous septa that frequently become incomplete – hallmarks of cirrhosis regression.[34] Reduced liver fibrosis was associated with, and very likely due to, HSC deactivation and decreased proliferation, as suggested by a marked decrease in α-SMA expression, and diminished desmin-positive HSCs. These effects are unique to carvedilol, and were not observed after propranolol administration, either *in vitro* or *in vivo*. The remarkable antifibrotic effects of carvedilol, and ensuing tissue remodeling and amelioration of liver structural abnormalities, undoubtedly play a major role in decreasing portal pressure *in vivo*. Deactivation of HSCs in cirrhosis has been observed previously with other drugs that antagonize LSEC dysfunction, such as simvastatin[35] and strong antioxidants such as recombinant human superoxide dismutase.[28,36,37,38] HSCs have been reported to be modulated by sympathetic neurotransmitters;[14] thus, we hypothesized and verified that the α1-adrenoceptor blocking activity of carvedilol contributes to blunting the contraction of activated HSCs after stimulation of α-1 adrenoceptors by methoxamine.

Our *in vivo* hemodynamic studies in rats with TAA-induced cirrhosis confirmed that carvedilol significantly reduced portal pressure, and more effectively than propranolol. This reduction was not accompanied by significant differences in the changes in mean arterial pressure, portal blood flow, or heart rate between these two drugs, thus pointing out that the additional portal pressure reduction achieved by carvedilol is not attributable to systemic hemodynamic effects but likely results from its beneficial effects on the intrahepatic circulation, primarily through two mechanisms: ameliorating sinusoidal endothelial dysfunction, thus blunting the increased liver vascular tone of cirrhosis, and through the deactivation of HSCs, thus decreasing liver fibrosis and improving liver structural abnormalities.

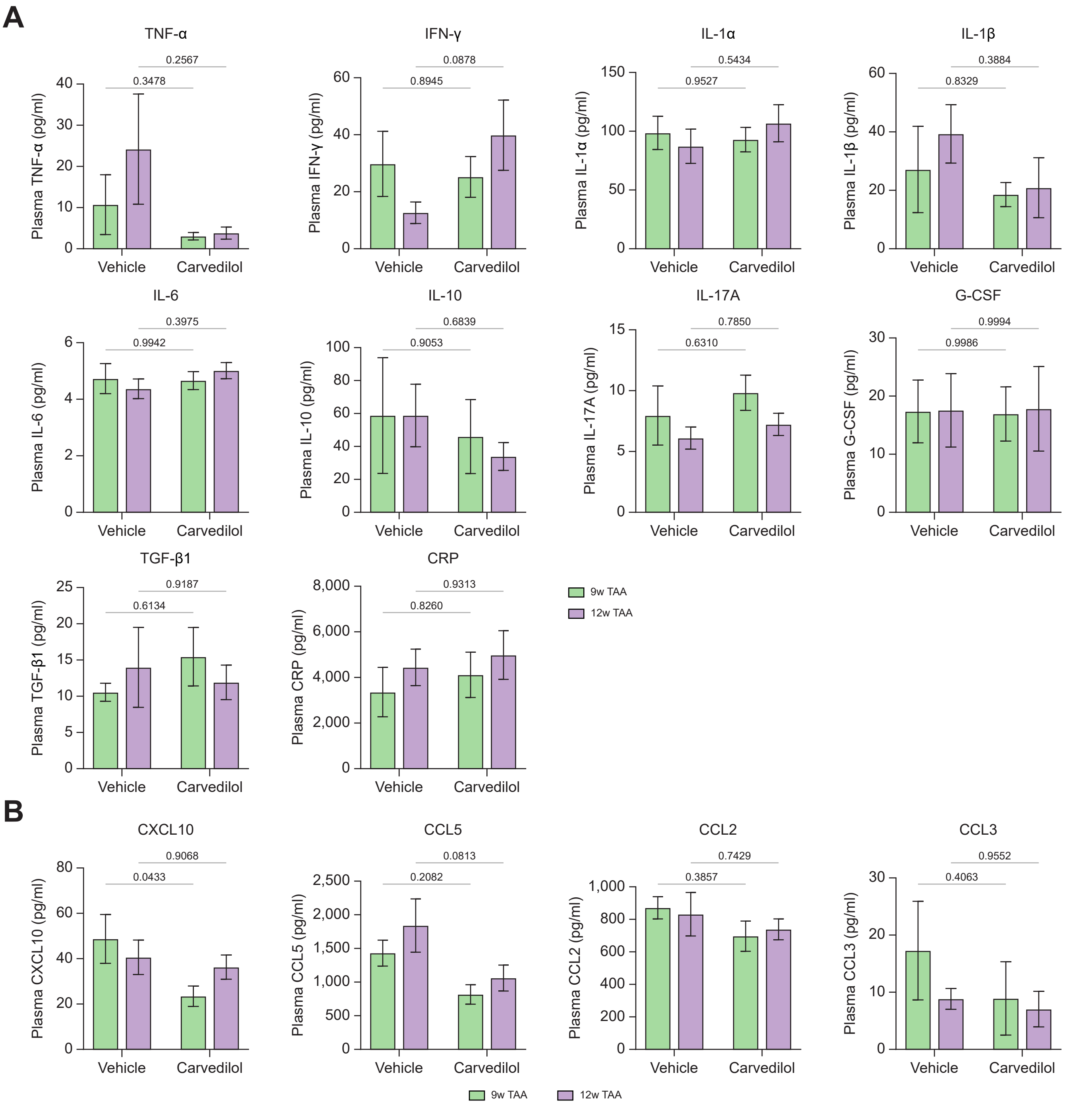

Fig. 8. Anti-inflammatory effects of carvedilol in rats with advanced and early cirrhosis. Expression of cytokines (A) and chemokines (B) in plasma from cirrhotic rats receiving vehicle or carvedilol (n = 6-9 per group). Mixed-effects analysis was performed. All data are represented as mean ± SEM. *p* values <0.05 were considered significant and are depicted in the figure. CCL2, chemokine ligand 2; CCL3, chemokine ligand 3; CCL5, chemokine ligand 5; CRP, C-reactive protein; CXCL10, C-X-C motif chemokine ligand 10; G-CSF, granulocyte colony-stimulating factor; IFN-γ, interferon-gamma; IL-1α, interleukin 1-alpha; IL-1β, interleukin 1-beta; IL-6, interleukin 6; IL-10, interleukin 10; IL-17A, interleukin 17A; TGF-β1, transforming growth factor beta 1; TNF-α, tumor necrosis factor.

Systemic inflammation plays a key role in the progression of chronic liver disease.[39] Importantly, carvedilol is believed to have anti-inflammatory effects.[40] We explored this by measuring plasma levels of cytokines and inflammatory mediators in cirrhotic rats. The pro-inflammatory factor TNF-α[41] was non-significantly reduced by carvedilol both in advanced and early cirrhotic rats, and data showed considerable variability. Importantly, there was an increase in IFN-γ, which has been reported to deactivate HSCs in a fibrotic rat model[42] and to abrogate pro-fibrogenic signaling *in vitro*.[43] A recent study identified IFN-γ as a protective factor in humans with cirrhosis, particularly in the decompensated stage.[44] Our findings are

consistent with this observation since carvedilol increased IFN-γ levels in advanced cirrhotic rats, but not in early cirrhosis. Among chemokines, carvedilol significantly reduced CXCL10 levels in early cirrhotic rats, but not in advanced cirrhosis, consistent with a previous study showing a link between increased CXCL10 and oxidative stress upregulation.[45] The stronger antioxidant effect of carvedilol in early cirrhotic rats compared to advanced cirrhotic rats may explain why carvedilol significantly reduced CXCL10 levels in early but not in advanced cirrhotic rats. Notably, CCL5 was the only chemokine that decreased in advanced and early cirrhosis, suggesting its potential as a therapeutic target for modulating inflammation across different stages of chronic liver disease.[46]

In conclusion, the present study indicates that the beneficial effects of carvedilol over propranolol are not limited to a greater reduction in portal pressure due to hemodynamic effects, but also involve marked improvements in the main intrahepatic determinants of portal hypertension in cirrhosis, including both structural and dynamic components. Carvedilol led to a marked reduction in liver fibrosis, associated with features of partial cirrhosis regression. These changes were accompanied by improvements in hepatic endothelial dysfunction, moderate anti-inflammatory effects, and improved liver function tests. These findings were further substantiated in human cells, underlining the translational potential of our results. Future long-term, longitudinal studies in human cirrhosis are warranted to verify the extent of these benefits, which may not only contribute to the superior efficacy of carvedilol over other drugs in preventing decompensation and improving survival, but also to promoting cirrhosis regression when combined with etiological therapy.

Affiliations

[1]*Department of Visceral Surgery and Medicine, Inselspital, Bern University Hospital, University of Bern, Switzerland;* [2]*Department for BioMedical Research, University of Bern, Switzerland;* [3]*Liver Vascular Biology Research Group, IDIBAPS Biomedical Research Institute, CIBEREHD, Spain*

Abbreviations

α1-AR, alpha-1 adrenoceptor; α-SMA, alpha smooth muscle actin; CCL5, chemokine (C–C motif) ligand 5; CPA, collagen proportionate area; CXCL10, C-X-C motif chemokine ligand 10; DAF-FM-DA, 4-amino-5-methylamino-2',7'-dichlorofluorescein diacetate; DHE, dihydroethidium; eNOS, endothelial nitric oxide synthase; HSC, hepatic stellate cell; HUVEC, human umbilical vein endothelial cell; IFN-γ, interferon-gamma; LSEC, liver sinusoidal endothelial cell; NO, nitric oxide; NSBB, non-selective β-blocker; O_2^-, superoxide; $ONOO^-$, peroxynitrite; TAA, thioacetamide; TNF-α, tumor necrosis factor-alpha; vWF, von Willebrand factor.

Financial support

This study was carried out at the Department for Biomedical Research at the University of Bern. This project was supported by own research funds of the Hepatology group, Department of Visceral Surgery and Medicine, Inselspital, Bern. Yeldos Nulan and Prof. Jaime Bosch received support from the Swiss Liver Foundation.

Conflict of interest

The authors declare no conflicts of interest pertaining to this manuscript.
Please refer to the accompanying ICMJE disclosure forms for further details.

Authors' contributions

Concept and design: JB, EF. Animal procedures: EF, YN, SES, MP. Experimental work: YN, EF, SES, MP. Validation: AB, JGS. Writing of article: YN, EF, JB.

Data availability

The data are available from the authors upon reasonable request.

Acknowledgements

This study was carried out at the Department for Biomedical Research at the University of Bern. The authors are indebted to Dr. Cong Wang, Philipp Kellmann, and Pauline Six for technical support.

Supplementary data

Supplementary data to this article can be found online at https://doi.org/10.1016/j.jhepr.2025.101681.

References

Author names in bold designate shared co-first authorship

[1] Tsochatzis EA, Bosch J, Burroughs AK. Liver cirrhosis. Lancet 2014;383(9930):1749–1761.

[2] Jacobs-Wingo JL, Espey DK, Groom AV, et al. Causes and disparities in death rates among Urban American Indian and Alaska Native Populations, 1999-2009. Am J Public Health 2016;106(5):906–914.

[3] Engelmann C, Claria J, Szabo G, et al. Pathophysiology of decompensated cirrhosis: portal hypertension, circulatory dysfunction, inflammation, metabolism and mitochondrial dysfunction. J Hepatol 2021;75(Suppl 1):S49–S66. Suppl 1.

[4] Felli E, Selicean S, Guixe-Muntet S, et al. Mechanobiology of portal hypertension. JHEP Rep 2023;5(11):100869.

[5] **Guixe-Muntet S**, **Quesada-Vazquez S**, Gracia-Sancho J. Pathophysiology and therapeutic options for cirrhotic portal hypertension. Lancet Gastroenterol Hepatol 2024;9(7):646–663.

[6] Gracia-Sancho J, Marrone G, Fernandez-Iglesias A. Hepatic microcirculation and mechanisms of portal hypertension. Nat Rev Gastroenterol Hepatol 2019;16(4):221–234.

[7] Iwakiri Y. Endothelial dysfunction in the regulation of cirrhosis and portal hypertension. Liver Int 2012;32(2):199–213.

[8] Reiberger T, Hofer BS. The Baveno VII concept of cirrhosis recompensation. Dig Liver Dis 2023;55(4):431–441.

[9] **Fuster-Martinez I**, **Calatayud S**. The current landscape of antifibrotic therapy across different organs: a systematic approach. Pharmacol Res 2024;205:107245.

[10] Bosch J, Garcia-Pagan JC. Prevention of variceal rebleeding. Lancet 2003;361(9361):952–954.

[11] Villanueva C, Albillos A, Bosch J, et al. Beta blockers to prevent decompensation of cirrhosis in patients with clinically significant portal hypertension (PREDESCI): a randomised, double-blind, placebo-controlled, multicentre trial. Lancet 2019;393(10181):1597–1608.

[12] Bosch J. Carvedilol for portal hypertension in patients with cirrhosis. Hepatology 2010;51(6):2214–2218.

[13] Dandona P, Ghanim H, Brooks DP. Antioxidant activity of carvedilol in cardiovascular disease. J Hypertens 2007;25(4):731–741.

[14] Oben JA, Roskams T, Yang S, et al. Hepatic fibrogenesis requires sympathetic neurotransmitters. Gut 2004;53(3):438–445.

[15] **Fernandez-Iglesias A**, **Ortega-Ribera M**, Guixe-Muntet S, et al. 4 in 1: antibody-free protocol for isolating the main hepatic cells from healthy and cirrhotic single rat livers. J Cell Mol Med 2019;23(2):877–886.

[16] Villanueva C, Albillos A, Genesca J, et al. Development of hyperdynamic circulation and response to beta-blockers in compensated cirrhosis with portal hypertension. Hepatology 2016;63(1):197–206.

[17] Wanless IR, Nakashima E, Sherman M. Regression of human cirrhosis. Morphologic features and the genesis of incomplete septal cirrhosis. Arch Pathol Lab Med 2000;124(11):1599–1607.

[18] Shahid A, Chen M, Lin C, et al. The beta-blocker carvedilol prevents benzo(a)pyrene-induced lung toxicity, inflammation and carcinogenesis. Cancers (Basel) 2023;15(3).

[19] Toyoda S, Haruyama A, Inami S, et al. Effects of carvedilol vs bisoprolol on inflammation and oxidative stress in patients with chronic heart failure. J Cardiol 2020;75(2):140–147.

[20] Amirshahrokhi K, Niapour A. Carvedilol attenuates brain damage in mice with hepatic encephalopathy. Int Immunopharmacol 2022;111:109119.
[21] Felli E, Nulan Y, Selicean S, et al. Emerging therapeutic targets for portal hypertension. Curr Hepatol Rep 2023;22(1):51–66.
[22] Bosch J. Carvedilol: the beta-blocker of choice for portal hypertension? Gut 2013;62(11):1529–1530.
[23] Sinagra E, Perricone G, D'Amico M, et al. Systematic review with meta-analysis: the haemodynamic effects of carvedilol compared with propranolol for portal hypertension in cirrhosis. Aliment Pharmacol Ther 2014;39(6):557–568.
[24] Albillos A, Krag A. Beta-blockers in the era of precision medicine in patients with cirrhosis. J Hepatol 2023;78(4):866–872.
[25] Villanueva C, Torres F, Sarin SK, et al. Carvedilol reduces the risk of decompensation and mortality in patients with compensated cirrhosis in a competing-risk meta-analysis. J Hepatol 2022;77(4):1014–1025.
[26] El-Demerdash E, Abdel-Sattar SA, El-Bakly WM, et al. Antifibrotic effects of carvedilol and impact of liver fibrosis on carvedilol pharmacokinetics in a rat model. Eur J Drug Metab Pharmacokinet 2017;42(5):767–779.
[27] Banares R, Moitinho E, Matilla A, et al. Randomized comparison of long-term carvedilol and propranolol administration in the treatment of portal hypertension in cirrhosis. Hepatology 2002;36(6):1367–1373.
[28] **Gracia-Sancho J**, **Lavina B**, Rodriguez-Vilarrupla A, et al. Increased oxidative stress in cirrhotic rat livers: a potential mechanism contributing to reduced nitric oxide bioavailability. Hepatology 2008;47(4):1248–1256.
[29] Cheng J, Kamiya K, Kodama I. Carvedilol: molecular and cellular basis for its multifaceted therapeutic potential. Cardiovasc Drug Rev 2001;19(2):152–171.
[30] Smith JS, Rajagopal S. The beta-arrestins: multifunctional regulators of G protein-coupled receptors. J Biol Chem 2016;291(17):8969–8977.
[31] Wisler JW, DeWire SM, Whalen EJ, et al. A unique mechanism of beta-blocker action: carvedilol stimulates beta-arrestin signaling. Proc Natl Acad Sci U S A 2007;104(42):16657–16662.
[32] Benkel T, Zimmermann M, Zeiner J, et al. How Carvedilol activates beta(2)-adrenoceptors. Nat Commun 2022;13(1):7109.
[33] **Lefkowitz RJ**, **Rockman HA**, Shim PJ, et al. How carvedilol does not activate beta(2)-adrenoceptors. Nat Commun 2023;14(1):7866.
[34] **Selicean S**, **Wang C**, **Guixe-Muntet S**, et al. Regression of portal hypertension: underlying mechanisms and therapeutic strategies. Hepatol Int 2021;15(1):36–50.
[35] **Marrone G**, **Maeso-Diaz R**, et al. KLF2 exerts antifibrotic and vasoprotective effects in cirrhotic rat livers: behind the molecular mechanisms of statins. Gut 2015;64(9):1434–1443.
[36] **Guillaume M**, **Rodriguez-Vilarrupla A**, Gracia-Sancho J, et al. Recombinant human manganese superoxide dismutase reduces liver fibrosis and portal pressure in CCl4-cirrhotic rats. J Hepatol 2013;58(2):240–246.
[37] Marrone G, Shah VH, Gracia-Sancho J. Sinusoidal communication in liver fibrosis and regeneration. J Hepatol 2016;65(3):608–617.
[38] Book WM. Carvedilol: a nonselective beta blocking agent with antioxidant properties. Congest Heart Fail 2002;8(3):173–177. 90.
[39] Koyama Y, Brenner DA. Liver inflammation and fibrosis. J Clin Invest 2017;127(1):55–64.
[40] **Alvarado-Tapias E**, **Brujats A**, Puente A, et al. Hemodynamic effects of carvedilol plus simvastatin in cirrhosis with severe portal hypertension and suboptimal response to beta-blockers: a double-blind, placebo-controlled, randomized-trial. Hepatology 2025;82(1):140–154.
[41] Tiegs G, Horst AK. TNF in the liver: targeting a central player in inflammation. Semin Immunopathol 2022;44(4):445–459.
[42] Baroni GS, D'Ambrosio L, Curto P, et al. Interferon gamma decreases hepatic stellate cell activation and extracellular matrix deposition in rat liver fibrosis. Hepatology 1996;23(5):1189–1199.
[43] Weng H, Mertens PR, Gressner AM, et al. IFN-gamma abrogates profibrogenic TGF-beta signaling in liver by targeting expression of inhibitory and receptor Smads. J Hepatol 2007;46(2):295–303.
[44] **Cao Z**, **Yao Y**, **Cai M**, et al. Blood markers for type-1,-2, and -3 inflammation are associated with severity of acutely decompensated cirrhosis. J Hepatol 2025;82(5):836–850.
[45] Zhang X, Shen J, Man K, et al. CXCL10 plays a key role as an inflammatory mediator and a non-invasive biomarker of non-alcoholic steatohepatitis. J Hepatol 2014;61(6):1365–1375.
[46] **Berres ML**, **Koenen RR**, Rueland A, et al. Antagonism of the chemokine Ccl5 ameliorates experimental liver fibrosis in mice. J Clin Invest 2010;120(11):4129–4140.

Keywords: Chronic liver disease; portal hypertension; non-selective beta blockers; NSBBs.

Received 17 March 2025; received in revised form 15 October 2025; accepted 12 November 2025; Available online 20 November 2025

Journal of Hepatology, Volume *8*

Supplemental information

Carvedilol decreases hepatic vascular resistance by reducing fibrogenesis and reversing endothelial dysfunction in cirrhotic rats

Yeldos Nulan, Eric Felli, Sonia-Emilia Selicean, Manuel Prampolini, Annalisa Berzigotti, Jordi Gracia-Sancho, and Jaume Bosch

Carvedilol decreases hepatic vascular resistance by reducing fibrogenesis and reversing endothelial dysfunction in cirrhotic rats

Yeldos Nulan, Eric Felli, Sonia-Emilia Selicean, Manuel Prampolini, Annalisa Berzigotti, Jordi Gracia-Sancho, Jaime Bosch

Table of contents

Supplementary methods…………………………………………………………….....2

Fig. S1……………………………………………………………………………….....6

Table S1……………………………………………………………………………..... 9

Table S2……………………………………………………………………………....10

Supplementary references……………………………………………………………… 11

Supplementary methods

Animal models of cirrhosis of the liver

Female and male Sprague-Dawley rats, were acquired and maintained under standard environmental conditions at the University of Bern animal facility. The conditions included a 12-hour light/dark cycle and 10-15 air exchanges per hour. Upon arrival, the rats were acclimatized for two weeks before starting any experiments or treatments. Throughout the study, the rats had free access to chow diet and water. All experimental procedures were approved by the Bern Cantonal Ethics Committee and adhered to the Laboratory Animal Care guidelines (approval number BE382022). The model of cirrhosis and portal hypertension (PH) was based on the twice weekly for a total of 12 weeks administration of thioacetamide (TAA) (Sigma-Aldrich; Ref. No. 172502) (200 mg/kg of body weight; intraperitoneally for 12 weeks). In addition, other groups of animals received TAA for only 9 weeks, which resulted in the development of an early cirrhosis stage. Before each administration, the rats' body weights were recorded, and the TAA dosage was adjusted accordingly. Animal welfare: Animals were regularly monitored for signs of distress or discomfort, and humane endpoints were established. All invasive procedures were carried out under appropriate anesthesia, and analgesia was administered as needed. All personnel were trained in proper animal handling.

Hepatic and systemic hemodynamics

Cirrhotic rats were administered either 10 mg/kg/day carvedilol (Hexal AG, Germany), 30 mg/kg/day propranolol (Ratiopharm, Germany), or water by oral gavage for two weeks. One hour after the final dose, the rats were anesthetized with inhaled isoflurane (Girovet: 469860). Anesthesia was 5% in induction and 2% in the maintenance period. Mean arterial pressure (MAP) was measured by cannulating the femoral artery, and portal pressure (PP) was measured by cannulating the mesenteric vein, both using a heparinized P50 catheter (Portex) connected to a pressure transducer. Portal blood flow (PBF) was determined using a non-constrictive perivascular ultrasonic transit-time flow probe (Transonic Systems Inc.) placed close to the liver hilus. Pressure and flow probes were connected to a PowerLab (4SP) data recording system, and data was displayed using LabChart v5.5.6 software. Hemodynamic parameters were recorded following a 20-minute stabilization period,

collecting stable data for 2 minutes. Temperature was regulated via a heating pad (temperature maintenance of 37°C; Physitemp TCAT-2LV) with live temp control with a rectal probe. After the hemodynamic study, blood samples were collected. Finally, the animals were euthanized with a pentobarbital overdose (200 mg/kg, Nembutal), and tissue samples were harvested for molecular and histological experiments.

Isolation of liver sinusoidal endothelial cells and pharmacological treatment

LSECs were plated on a plastic plate (collagen type I pre-coated) and allowed to attach for 40 mins. After two washes with DPBS, LSECs were treated with a 10 μM propranolol, 10 μM carvedilol, or vehicle (dimethylsulfoxide (0.1%), Sigma) for another 24 h. The isolation protocol was generated by Fernández-Iglesias et.al. (1). Rats were intraperitoneally anesthetized with a combination of ketamine (100 mg/kg) and midazolam (5 mg/kg), with doses reduced by 30% for cirrhotic rats. The livers were perfused with a modified Hank's Buffer, followed by two collagenase A digestion steps: performed in situ and after liver excision. The resulting liver suspension was filtered into an ice-cold buffer through a 100 μm nylon strainer. It was then centrifuged at 60g for 5 minutes, yielding a pellet containing hepatocytes and a supernatant with non-parenchymal cells (NPCs). NPCs were further separated via a three-step OptiPrep iodixanol gradient centrifugation (0%, 13%, and 17%) into hepatic stellate cells (HSCs) and a fraction containing liver sinusoidal endothelial cells (LSECs) and Kupffer cells (KCs). LSECs were then isolated from KCs by exploiting non-specific adhesion.

Cell culture

Freshly isolated LSECs were cultured in RPMI 1640 Medium (ThermoFisher: 21875034) supplemented with 10% Fetal Bovine Serum (FBS, Gibco: A4766801), 1% antibiotic-antimycotic (Sigma: A5955), 50 μg/mL endothelial cell growth supplement (ECGS, Merck: 02-102), and 100 μg/mL heparin (Sigma: H-3393). Freshly isolated HSCs were maintained in Iscove's Modified Dulbecco's Medium (IMDM, ThermoFisher: 12440053) with 10% FBS and 1% antibiotic-antimycotic. HUVECs, purchased from Lonza (C2519A, Switzerland), were cultured in Medium 199 (Sigma-Aldrich: M4530) containing 20% FBS, 1% antibiotic-antimycotic, 50 μg/mL ECGS, and 100 μg/mL heparin. The LX-2 human hepatic stellate cell line was obtained from the Liver Vascular Biology Research Group, IDIBAPS Biomedical Research Institute and cultured in Dulbecco's Modified Eagle's Medium/Nutrient Mixture F-12 (DMEM, Gibco:

11330032) with 10% FBS and 1% antibiotic-antimycotic. All cell types were incubated at 37°C in a humid atmosphere (95%) with 5% CO_2.

Metabolic activity of Carvedilol and Propranolol in vitro

Cells in the exponential growth phase were seeded into 96-well plates with 100 µl of media per well and incubated for 24 hours. Subsequently, the cells were treated with various concentrations of carvedilol (0–100 µmol; Sigma: C3993) or propranolol (0–1000 µmol; Selleckchem: S4076) for 24 hours. After treatment, wells were gently washed twice with sterile PBS to remove residual drug and serum that could interfere with the assay. On the third day, 10 µl of MTT solution (5 mg/ml) was added to each well and incubated for 4 hours. Following incubation, the supernatant was carefully removed, and 100 µl of dimethylsulfoxide (DMSO; Sigma, Cat. No. 41647) was added to dissolve the formazan crystals. The DMSO concentration was maintained at 0.1%, and the total reagent volume was optimized for compatibility with our plate reader. After 15 minutes of incubation at room temperature in the dark, absorbance was measured at 595 nm using a BioTek Microplate Spectrophotometer (data not shown).

Cell proliferation assay

The proliferation profile of the human hepatic stellate cell line (LX2) was evaluated by the 5-bromo-2'-deoxyuridine (BrdU) incorporation assay. Briefly, cells were cultured in a 6-well plate with 1 ml media for 24 h. After that, cells were treated with or without carvedilol (10 µM) for 24 h. The culture medium was replaced with the BrdU labeling solution (Thermo: B23151) for 2 h in the incubator, followed by washing twice with PBS. Then, cells are fixed with 4% paraformaldehyde (PFA) for 15 mins at RT and washed three times with PBS. Permeabilization was achieved by adding 1ml 0.1% triton X-100 (Sigma) in RT for 20 mins. Subsequently, cells were treated with 1 ml of 1N hydrochloric acid (HCL) for 10 mins on ice, followed by 1 ml of 2N HCl for 10 mins at RT. Acid treatment was neutralized by incubating the cells with 1 ml of phosphate/citric acid buffer (pH 7.4, 182 mL of 0.2 M Na_2HPO_4 + 18 mL 0.1 M citric acid) for 10 mins at RT. The cells were washed three times with 0.1% triton X-100 (Sigma). To detect incorporated BrdU, cells were incubated with 1ml of BrdU antibody (Thermo: MA3-071, 1:100) at 4°C overnight. After washing three times with 0.1% triton X-100 (Sigma), cells were incubated with a fluorescently labeled secondary antibody for 1 hour at RT (Thermo: A21422, 2µg/ml). The cells were washed with PBS and then

for imaging in EVOS 5000 with a 10× objective in the Red Fluorescent Protein (RFP) channel. BrdU-positive cells were discriminated over DAPI channels and normalized with the Vehicle group. Quantification was made with ImageJ. Cell seeding density was 30,000/cm^2.

Cell contraction

Cell contraction assays of HSCs and LX2 cells were done in collagen lattices (collagen I rat tail; ThermoFisher: A1048301). Briefly, freshly isolated HSCs were cultured in a plastic flask for 5 days to reach a fully activated morphology. Then, 24-well culture plates were pre-incubated with 1% Bovine Serum Albumin (BSA, Sigma: A7906) in PBS, after which they were filled with a mixture of collagen I (1.3 mg/ml) and HSCs (1.5×10^5 cells/ml). After collagen gel solidification, serum-free media containing 10 μM carvedilol or propranolol was added for 24 h incubation. Cell contraction was induced by adding 10% FBS for 48 h and photographed at 1, 24, and 48 h. In another set of experiments, 500 μM of the α1-adrenergic receptor agonist methoxamine (Merck: M6524) was used to accelerate the cell contraction and the inhibitory activity of carvedilol and propranolol (both 10 μM) was analyzed in photographs at 1 h.

Superoxide detection

Superoxide (O^{2-}) levels were assessed using the oxidative fluorescent dye Dihydroethidium (DHE, 10 μM, Sigma, D7008) in liver-frozen sections, HUVECs, and LX2 cells. Cirrhotic liver tissue frozen at -80°C was sectioned into 10-micrometer slices using the Leica Cryostat CM1950 system. The tissue sections were loaded with 10 μM DHE and incubated at RT for 30 minutes. Slides were then imaged with fluorescent aqueous mounting medium (Dako; S302380-2). For the cell lines, the culture medium was removed, and the cells were rinsed twice with PBS. Subsequently, 10 μM DHE was added, and the cells were incubated for 30 minutes in an incubator. After incubation, the dye solution was replaced with PBS, and images were immediately captured. All fluorescent images were acquired using an EVOS 5000 microscope with a 10x objective. Quantitative analysis of the fluorescent signal was performed using ImageJ software version 1.53.

Immunostaining

Liver tissue for immunohistochemistry was fixed in 4% formaldehyde (Sigma), embedded in paraffin, and sectioned. Dewaxing the tissue with Xylene (2 times,

5mins) and series ethanol dilution. Heated-EDTA-Tris buffer (PH=9) was used to reveal the antigen and rinsed with distilled water two times. Unspecific binding was blocked with 3% goat serum for 1 h. Liver sections were incubated with primary antibody against alpha-smooth muscle actin (α-SMA) (Thermo; 14976082; 1:400) to mark the activated hepatic stellate cells at 4°C overnight. Secondary antibody incubation was performed with Goat anti-Mouse IgG H&L Alexa Fluor® 555 (Thermo; A21422; 2μg/ml) in combination with 4′,6-diamidino-2-phenylindole (DAPI) for 1 h at RT. Conjugated desmin (Thermo; eFluor™ 660; 50-9747-82; 10μg/ml) was used to detect proliferating hepatic stellate cells at 4°C overnight and then in combination with DAPI for 1 h at RT. Activated endothelial cells were marked by von Willebrand factor (vWF) (Thermo; PA5-80223; 2μg/ml) at 4°C overnight. Secondary antibody incubation was performed with Goat anti-Rabbit IgG H&L Alexa Fluor® 488 (Abcam; 150077; 1:500) in combination with DAPI for 1 h at RT. All slides were then mounted with fluorescent aqueous mounting medium (Dako; S302380-2) and dried overnight. Then sections were captured using a 3DHistech Slide scanner, (Panoramic 250 Flash II). The α-SMA stained areas were quantified using Qupath-0.4.3. Desmin and vWF positive stained cell numbers were quantified using Qupath-0.4.3.

Western Blot

Cells on the culture plate were lysed in RIPA Buffer. Protein concentrations were determined using the Pierce™ BCA Protein Assay Kit (Thermo #23225). Protein extracts were boiled in RIPA buffer (Sigma #R0278) and reducing sample buffer (Thermo #39000) at 95 °C for 10 min. A total of 15 μL samples (10ug of protein/lane) were separated by sodium dodecyl sulfate-polyacrylamide gel electrophoresis (SDS-PAGE) using Bolt™ Bis-Tris Plus Mini Protein Gels, 4-12%, 1.0 mm, WedgeWell™ format 12 wells (Thermo #NW04125BOX). Proteins were then transferred onto PVDF membranes, blocked with 5% BSA in TBS-T for 60 min at room temperature, and washed 1 time with TBST. Primary antibody against eNOS polyclonal antibody (Thermo, PA1-037, 1:1000) incubated overnight at 4 °C and 1 hour at RT with HRP-conjugated Goat anti-Rabbit IgG (H+L) Cross-Adsorbed Secondary Antibody (Invitrogen, G21234, 1:1000). Primary antibody against phosphor-eNOS (Ser1177) polyclonal antibody (Thermo, PA5-104858, 1:1000) incubated overnight at 4 °C and 1 hour at RT with HRP-conjugated Goat anti-Rabbit IgG (H+L) Cross-Adsorbed Secondary Antibody (Invitrogen, G21234, 1:1000). Primary antibody against

nitrotyrosine (Abcam, ab42789, 1:250) incubated overnight at 4 °C and 1 hour at RT with HRP-conjugated Goat anti-Rabbit IgG (H+L) Cross-Adsorbed Secondary Antibody (Invitrogen, G21234, 1:1000). HRP conjugated anti-GAPDH antibody (Abcam, #201822, 1:10000) was used for loading control.

In liver tissue (same preparation as described above), a total of 15 μL samples (10ug of protein/lane) were separated by SDS-PAGE using Bolt™ Bis-Tris Plus Mini Protein Gels, 4-12%, 1.0 mm, WedgeWell™ format 12 wells (Thermo #NW04125BOX). Proteins were then transferred onto PVDF membranes. The blotted membranes were immersed in 1X iBind™ Flex Solution (Thermo Scientific #SLF1020) for 10 mins at RT. The immunodetection was done using iBind Flex Western System (Thermo Scientist #SLF1000). The membrane was incubated 2.5 h at RT with primary antibody against α-SMA (Thermo Scientific, #14-9760-82, 1:100), and then with HRP-conjugated Goat Anti-Mouse IgG & IgM Antibody (Merck, AP130P, 1:400). HRP Anti-GAPDH antibody (Abcam, ab201822, 1:10000) as a loading control. All the membrane was incubated with chemiluminescence detection reagents and visualized by the Fusion FX system.

Systemic Inflammation Multiplex Immunoassay

Plasma from cirrhotic rats was collected and analyzed using the ProcartaPlex Immunoassay (Thermo). ProcartaPlex assays are multiplex immunoassays that utilize antibody-coated magnetic beads and are performed in the Bio-Plex 3D Suspension Array system, enabling the simultaneous detection and quantification of multiple cytokines and chemokines. Following hemodynamics measurements, rat blood was collected from the inferior vena cava into EDTA K3E-coated tubes and centrifuged (800 RCF, 20 mins, 4°C) to obtain plasma. Later, Plasma samples were centrifuged at 10,000 × g for 10 minutes to remove particulates. Subsequently, 25 µl of plasma was diluted with 25 µl of Universal Assay Buffer (Thermo). 25 µl of dilution was incubated in a customized ProcartaPlex plate for 2 h with shaking at RT and then washed three times. Subsequently, 25 µl Detection Antibody Mix was added, shaken at RT for 30 mins, and washed three times. Later, add 50 µl of Streptavidin-PE solution, shaking at RT for 30 mins with three times washing. In the end, 120 µl Reading Buffer was added and the plate was shaken at RT for 5 mins. Data was acquired on the Bio-Plex 3D Suspension Array system and analyzed using Bio-Plex Manager Software 6.0.

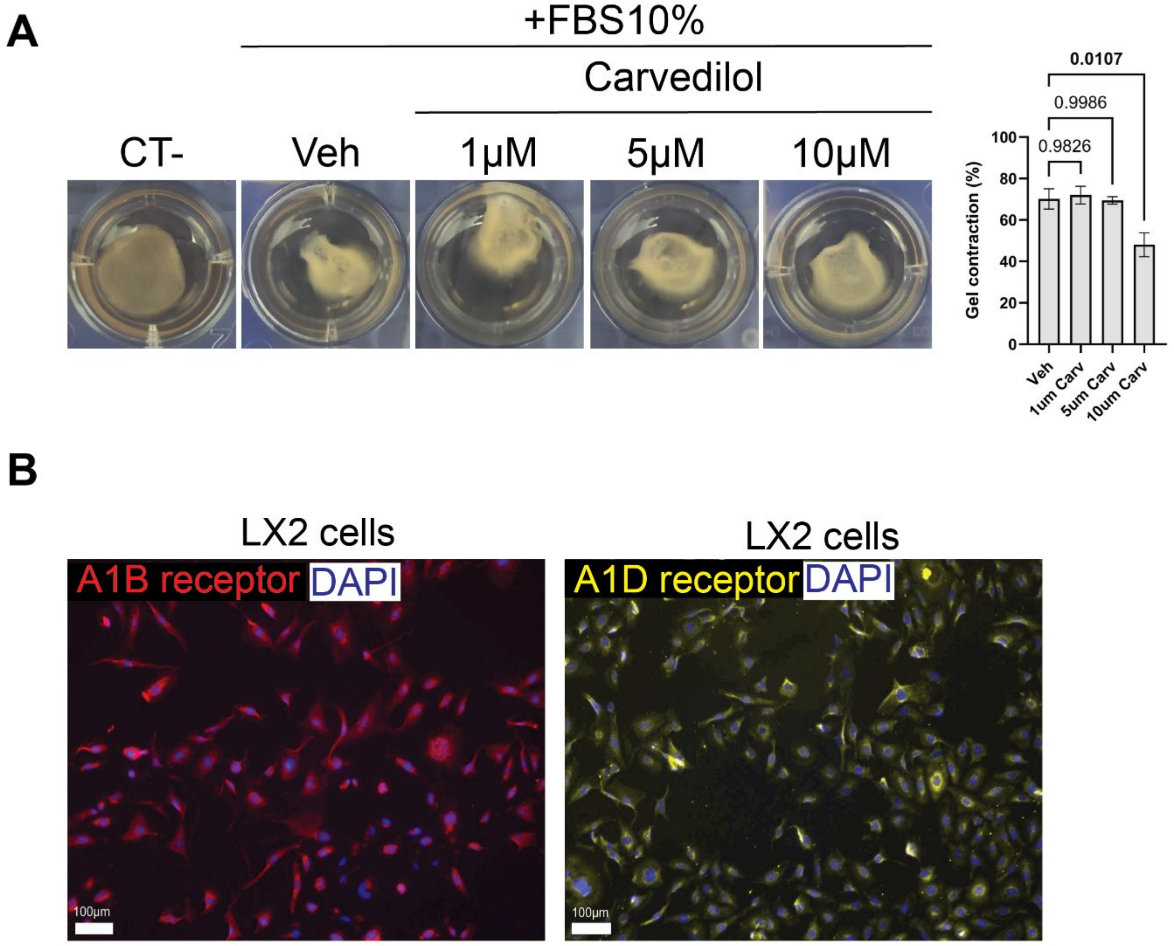

Fig. S1. Carvedilol inhibits human hepatic stellate cells contraction. A) Cell contraction assay in response to different concentrations of Carvedilol in LX2 cells. N=4. Sample distributions were assessed for normality (Kolmogorov−Smirnov test). Normally distributed data were compared to the vehicle with one-way ANOVA (multiple comparison Tukey). All data are represented as mean ± sem. P-value <0.05 was considered significant and depicted in the figure. B) alpha1B-adrenoceptor and alpha1D-adrenoceptor expression verified on LX2 cells. Representative images were captured using the EVOS 5000 system, 20x objective. N=3.

	Vehicle	Propranolol	Carvedilol
**ALT (U/l)**	68.7 ± 5.0	70.2 ± 6.2	62.4 ± 3.1
**AST (U/l)**	89.3 ± 10.5	101.0 ± 10.4	86.4 ± 6.9
**Bilirubin (µmol/l)**	3.1 ± 0.1	3.1 ± 0.2	2.9 ± 0.0
**Creatinine (µmol/l)**	58.9 ± 9.2	42.5 ± 9.4	41.8 ± 2.0
**Total Protein (g/l)**	52.8 ± 0.9	48.9 ± 0.7	48.9 ± 1.7
**Liver weight (gr)**	14.6 ± 1.3	14.0 ± 0.7	14.3 ± 1.3
**Body weight (gr)**	393.4 ± 29.4	389.0 ± 13.9	391.6 ± 26.4
**Spleen weight (gr)**	1.1 ± 0.1	1.2 ± 0.1	1.2 ± 0.1
**n**	8	6	8

Table S1. Blood analysis and corresponding organ weight from 12-week TAA cirrhotic rats treated with vehicle, propranolol or carvedilol. Kruskal−Wallis followed by Dunn's multiple comparisons were performed. All data are represented as mean ± sem. All data are represented as mean ± sem. P-value <0.05 was considered significant. No statistical significance were found.

Comparison of carvedilol effects in advanced and early cirrhosis rats

	Advanced cirrhosis (TAA 12w)			Early cirrhosis (TAA 9w)		
Parameters	Vehicle	Carvedilol	Relative ratio (Carv/Veh)	Vehicle	Carvedilol	Relative ratio (Carv/Veh)
Number of animals	8	8	/	9	10	/
PP (mmHg)	20.0 ± 1.7	15.4 ± 2.8*	- 22%	16.7 ± 1.5	14.0 ± 1.9*	-17%
PBF/BW (ml/min/100gr)	5.1 ± 1.1	4.1 ± 0.8	- 19%	4.8 ± 1.2	4.2 ± 1.0	- 13%
MAP (mmHg)	96 ± 6	84 ± 12	- 12%	94 ± 9	82 ± 7*	- 13%
Heart rate (Bpm)	274 ± 26	249 ± 35	- 9%	296 ± 26	255 ± 20*	- 14%
Liver weight (g)	14.7 ± 3.8	14.3 ± 3.7	- 0.6%	14.0 ± 2.9	14.0 ± 3.6	+ 0.1%
Body weight (g)	393 ± 83	392 ± 75	- 0.4%	369 ± 87	383 ± 95	+ 3%
CPA (%)	19.8 ± 2.9	14.6 ± 2.7*	- 26.8%	17.0 ± 1.9	13.1 ± 3.1*	- 23.1%
α-SMA (%)	3.8 ± 0.5	2.9 ± 0.4*	- 22.7%	3.5 ± 0.5	2.9 ± 0.6*	- 17.4%
Desmin (number)	7 ± 1	5 ± 1*	- 29.8%	6 ± 1	5 ± 1*	- 20.4%
vWF (number)	6 ± 1	5 ± 1*	- 26.3%	5 ± 1	4 ± 1*	- 16.9%
DHE (intensity)	1 ± 0.2	0.7 ± 0.3	- 26.1%	1 ± 0.3	0.5 ± 0.2*	- 52.9%

NOTE. Values represent mean ± standard deviation.

TAA, thioacetamide; PP, portal pressure; PBF, portal blood flow; BW, body weight; MAP, mean arterial pressure; CPA, collagen proportionate area; α-SMA, alpha-smooth muscle actin; vWF, Von Willebrand factor; DHE, dihydroethidium

*P < 0.05 vs vehicle.

Table S2. Comparison of carvedilol effects in advanced and early cirrhosis rats. Sample distributions were assessed for normality (Kolmogorov−Smirnov test). Normally distributed data were compared with unpaired Student t-test otherwise with Mann−Whitney test. All data are represented as mean ± s.d. *P <0.05 was considered significant.

Supplementary reference

1. **Fernández-Iglesias A, Ortega-Ribera M**, Guixé-Muntet S, Gracia-Sancho J. 4 in 1: Antibody-free protocol for isolating the main hepatic cells from healthy and cirrhotic single rat livers. J Cell Mol Med. 2019;23(2):877-86.
